# First-in-human phase I study of ISTH0036, an antisense oligonucleotide selectively targeting transforming growth factor beta 2 (TGF-β2), in subjects with open-angle glaucoma undergoing glaucoma filtration surgery

**DOI:** 10.1371/journal.pone.0188899

**Published:** 2017-11-30

**Authors:** Norbert Pfeiffer, Bogomil Voykov, Giulia Renieri, Katharina Bell, Paul Richter, Melanie Weigel, Hagen Thieme, Barbara Wilhelm, Katrin Lorenz, Martin Feindor, Katja Wosikowski, Michel Janicot, Daniela Päckert, Regina Römmich, Carola Mala, Petra Fettes, Eugen Leo

**Affiliations:** 1 Dpt. of Ophthalmology, University Medical Center Mainz, Mainz, Germany; 2 Dpt. of Ophthalmology, University Hospital Tuebingen, Tuebingen, Germany; 3 Dpt. of Ophthalmology, Otto-von-Guericke-University, Magdeburg, Germany; 4 STZ Eyetrial, University Hospital Tuebingen, Tuebingen, Germany; 5 SynteractHCR Deutschland GmbH, Munich, Germany; 6 Isarna Therapeutics GmbH, Munich, Germany; Universita degli Studi di Firenze, ITALY

## Abstract

**Purpose:**

To evaluate the safety and tolerability of intravitreal ISTH0036, an antisense oligonucleotide selectively targeting transforming growth factor beta 2 (TGF-β2), in patients with primary open angle glaucoma (POAG) undergoing trabeculectomy (TE; glaucoma filtration surgery).

**Methods:**

In this prospective phase I trial glaucoma patients scheduled for TE with mitomycin C (MMC) received a single intravitreal injection of ISTH0036 at the end of surgery in escalating total doses of 6.75 μg, 22.5 μg, 67.5 μg or 225 μg, resulting in calculated intraocular ISTH0036 concentrations in the vitreous humor of approximately 0.3 μM, 1 μM, 3 μM or 10 μM after injection, respectively. Outcomes assessed included: type and frequency of adverse events (AEs), intraocular pressure (IOP), numbers of interventions post trabeculectomy, bleb survival, visual acuity, visual field, electroretinogram (ERG), slit lamp biomicroscopy and optic disc assessment.

**Results:**

In total, 12 patients were treated in the 4 dose groups. Main ocular AEs observed were corneal erosion, corneal epithelium defect, or too high or too low IOP, among others. No AE was reported to be related to ISTH0036. All other safety-related analyses did not reveal any toxicities of concern, either. The mean medicated preoperative IOP at decision time-point for surgery was 27.3 mmHg +/- 12.6 mmHg (SD). Mean IOP (±SD) for dose levels 1, 2, 3, and 4 were at Day 43 9.8 mmHg ± 1.0 mmHg, 11.3 mmHg ± 6.7 mmHg, 5.5 mmHg ± 3.0 mmHg and 7.5 mmHg ± 2.3 mmHg SD; and at Day 85 9.7 mmHg ± 3.3 mmHg, 14.2 mmHg ± 6.5 mmHg, 5.8 mmHg ± 1.8 mmHg and 7.8 mmHg ± 0.6 mmHg, respectively. In contrast to IOP values for dose levels 1 and 2, IOP values for dose levels 3 and 4 persistently remained below 10 mmHg throughout the observation period.

**Conclusion:**

This first-in-human trial demonstrates that intravitreal injection of ISTH0036 at the end of TE is safe. Regarding IOP control, single-dose ISTH0036 administration of 67.5 μg or 225 μg at the time of TE resulted in IOP values persistently < 10 mmHg over the three month postoperative observation period.

## Introduction

Glaucoma currently affects more than 70 million people worldwide [1] and is the second leading cause for irreversible blindness in the Western world [2]. The disease is characterized by optic nerve head damage, retinal ganglion cell death and progressive visual field loss. In the majority of cases increased IOP is present and appears to be a main contributing pathophysiologic factor. At present existing pharmacologic treatments are directed mainly towards the lowering of IOP.

The majority of patients is treated with IOP lowering therapy. However, if disease progresses in spite of maximally tolerated therapy surgery may be indicated. Trabeculectomy (TE; glaucoma filtration surgery) is one of the most frequent surgical interventions and allows for drainage of aqueous from the anterior chamber of the eye to the subconjunctival space. Despite alternative techniques and devices emerging, TE remains the most frequent procedure for IOP lowering worldwide [3, 4]. However, success of TE is endangered by postoperative scarring, resulting in fibrotic closure of the drainage and rising IOP [5, 6]. To prevent this scarring of conjunctival tissue and the Tenon’s capsule, currently antimetabolites such as MMC and 5-FU are used but are accompanied by unwanted side-effects [7, 8]. Delayed wound healing may even lead to blebitis, choroidal detachments, conjunctival dehiscence, bleb leakage, long-term hypotony [9] and dysesthesia [7, 8].

Transforming growth factor beta 2 (TGF-β2) has been linked to the main pathophysiologic events in glaucoma: (1) trabecular meshwork alteration by epithelial-to-mesenchymal transition resulting in rise of IOP is seen as driven by it [10], and (2) optic nerve head damage by tissue remodeling [11, 12]. Glaucoma patients not only have substantially elevated levels of TGF-β2 [13–16] but the optic nerve head as area of main glaucoma damage contains 70–100 fold elevated levels of TGF-β2 [11]. In addition, TGF-β2 also has been identified as a core driver of intraocular fibrosis [17–20], and specifically has been linked to fibrotic overgrowth following TE. Consequently, it is for several reasons of high interest to investigate the safety and efficacy of TGF-β2 targeting agents in glaucoma and specifically the TE setting. Ultimately, this may not only increase the success rate of glaucoma filtration surgery but may improve outcomes in glaucoma by providing optic nerve protection and preventing further alteration of the trabecular meshwork.

This study is the first-in-human trial of the locked nucleic acid technology modified 14-mer fully phosphorothioate antisense oligodeoxynucleotide ISTH0036 that selectively targets the TGF-β2 isoform. Preclinical data indicated favorable pharmacokinetic and pharmacodynamic properties with potent and selective long-term suppression of the target in in-vitro and in-vivo assays. In addition, ISTH0036 showed potent anti-fibrotic and antiangiogenic effects in a choroidal neovascularization in-vivo model and preserved bleb size and survival in a glaucoma filtration mouse model when administered intravitreally [21–23].

## Materials and methods

### Trial design and conduct

This prospective phase I, first-in-human, open-label, dose-escalation trial (EudraCT# 2014-004985-74, https://eudract.ema.europa.eu, and NCT02406833 on https://www.clinicaltrials.gov) was conducted between April 2015 and August 2016 at three trial sites (Department of Ophthalmology of Mainz University Medical Center, University Hospital Tuebingen and Otto-von-Guericke University Magdeburg, Germany). Objective of the study was to evaluate the safety and tolerability as well as to observe preliminary clinical efficacy of single intravitreal injections of ISTH0036 at the time of TE. The study was approved by the Ethics Committee of the State Chamber of Medicine Rhineland Palatinate, Mainz and the German Federal Institute for Drugs and Medical Devices, BfArM and was conducted in accordance with the Declaration of Helsinki.

Main inclusion criteria were age 18–80 years, a diagnosis of primary open-angle glaucoma (high tension or normal tension) with patients scheduled for TE due to not tolerating medical therapy or progressing in spite of maximally tolerated medical therapy. Exclusion criteria were history of any other form of glaucoma in either eye, history of relevant ocular trauma in either eye < 6 months, history of ocular infection or ocular inflammation in either eye < 3 months. Patients underwent a washout-period according to their treating surgeons’ standards (usually 4 weeks for topical beta-blockers and prostaglandins, 7 days for topical alpha-2-agonists and carbonic anhydrase inhibitors, and at least 14 days for any ophthalmic medication or substance which had not been taken at a stable dose).

Subjects received TE with topical MMC (100 μl of 0.2 mg/ml solution) and a single intravitreal injection of ISTH0036 at the end of the surgical procedure. Three subjects per dose level (DL) were enrolled, receiving escalating total intravitreal doses of 6.75 μg, 22.5 μg, 67.5 μg or 225 μg (injection volume 50 μl), respectively, resulting in calculated intraocular ISTH0036 concentrations in the vitreous humor of approximately 0.3 μM, 1 μM, 3 μM or 10 μM. The starting dose of 6.75 μg reflects 1/10^th^ of the preclinical No Observed Adverse Effect Level (NOAEL) from a 4-week intravitreal rabbit toxicology study. In preclinical cell-based experiments, ISTH0036 potently and specifically suppressed TGF-β2 mRNA and protein with IC_50_ values of 0.4 and 0.7 μM, respectively, demonstrating that sub-micromolar concentrations were pharmacodynamically effective [24]. The upper limit of dose escalation was set based upon observed toxicological findings at higher doses. There was a minimum interval of 1 week between the ISTH0036 dosing of the first and all subsequent subjects in each cohort as an additional safety measure. Dose escalation was carried out when data from 3 subjects who had completed the dose limiting toxicity (DLT) monitoring period of 42 days were available and had been reviewed by the Cohort Review Committee and no DLT had been observed.

Standard concomitant medications during the post-operative phase were topical antibiotics, topical glucocorticoids, and topical atropine or equivalent. Depending on bleb status and if deemed necessary by the Investigator subconjunctival administration of 5-Fluorouracil (5-FU at 5 mg/0.5 ml) and topical anti-glaucoma medications (e.g. beta-blockers, alpha-2-agonists etc.) were permitted, as well as non-pharmacological interventions (e.g. needling, suture lysis or needling procedure) and were documented as post-trabeculectomy interventions.

Primary endpoint was type and frequency of AEs (AE reporting according to National Cancer Institute (NCI) Common Terminology Criteria for Adverse Events (CTCAE) v4.03) for the reporting period. Slit-lamp and fundus examination, vital signs and safety laboratory were performed at various timepoints throughout the study. An ERG was conducted at screening and at the end of the DLT period (week 6) to monitor retinal function. DLTs were defined as all toxicities observed during the 42 days DLT period following the intravitreal injection that are at least possibly related to ISTH0036 and are NCI CTCAE v4.03 AE ≥ Grade 3 or eye disorder AE ≥ Grade 2 or cataract, retinal detachment, retinopathy ≥ Grade 1. Slit lamp deterioration of two grades or more had to be reported as AE. Secondary endpoints were IOP at the end of the study, number of interventions post trabeculectomy, bleb filtering and bleb morphology, best corrected visual acuity, visual field, slit lamp biomicroscopy, and optic disc status as determined by biomorphometry of the optic disc Heidelberg Retinograph II (HRTII) and by photography of the optic disc. IOP was measured at baseline, week 6 and week 12 in both eyes by Goldmann applanation tonometry (in sitting position with same fluorescein and anesthetic agents at each measurement). For each eye, the mean of two readings or the median of three readings in case of differences > 2 mmHg were recorded for the analysis using the same calibrated Goldmann applanation tonometer throughout the study in a patient. Bleb morphology and bleb filtering was assessed by using slit lamp images on Day 3, week 6 and week 12. The bleb was classified using the Wuerzburg Bleb Classification Score [25]. Best corrected visual acuity (BCVA) was measured for both eyes under dim room light using Early Treatment Diabetic Retinopathy Study (ETDRS) charts and the logMAR scoring system (baseline, Day 3, week 6 and week 12). Visual field measurement took place at baseline, week 6 and week 12 using Humphrey or Octopus Standard 24–2 or 30–2 white on white perimetry. The eyelids, conjunctiva, cornea, iris/anterior chamber and lens were examined at baseline, week 6 and week 12 by slit lamp biomicroscopy. The stereometric parameters of the optic disc were measured on week 6 and week 12 by confocal scanning laser ophthalmoscopy with Heidelberg Retina Tomograph II (HRTII). The Moorefield’s Regression Analysis and the Topographic Change Analysis were used to detect progression. Optic disc photographs were taken to evaluate changes in the optic disc.

### Investigational agent

ISTH0036 is a synthetic 14-mer fully phosphorothioate antisense oligodeoxynucleotide modified with locked nucleic acids [26] (3+3 LNA-modified gapmer) selectively targeting the messenger ribonucleic acid (mRNA) of the TGF-β2 isoform. Potent, selective, sequence- and dose-dependent downregulation of TGF-β2 mRNA and protein was detected in human A172, Panc1 and trabecular meshwork cells as well as in murine astrocytes treated with different concentrations of ISTH0036 [21–24]. In addition, decreased TGF-β2 mRNA and protein levels in ocular tissues were observed after a single intravitreal administration of ISTH0036 to New Zealand White rabbit eyes for up to 8 weeks duration [24]. The drug product contains 6.75 mg anhydrous ISTH0036 sodium salt/vial expressed as total oligonucleotide content. Immediately prior to administration, the lyophilized powder is reconstituted aseptically in isotonic (0.9%) saline solution to 50 μl injection volume in total.

### Data review and protocol deviations

After closure of the database a review of data according to the ICH-guideline E9 was conducted in order to classify the whole patient population into two analysis sets by applying the corresponding criteria (full analysis set [FAS] reflective of all subjects included who completed at least the follow-up visit 6 weeks post injection and safety analysis set [SAS] reflective of all subjects that received at least one dose of study medication) and to also decide whether a protocol deviation is major or minor. 36 minor and one major protocol deviation were identified/reported for patients treated in this study. The minor protocol deviations were deviations from the scheduled timing of assessments or missed assessments. The one major protocol deviation occurred when a safety-relevant follow-up visit assessment was performed in error on Day 42 instead of Day 43 (one day before formal completion of the DLT period). None of the patients were excluded from the full analysis set (FAS) due to protocol deviations. FAS and SAS were identical populations.

### Statistical analysis

For this phase I study no inferential statistical hypothesis was created, and descriptive analyses were conducted, only, using SAS^®^ Statistical Analysis System, Version 9.3 or higher.

## Results

### Patient population

**Fig 1:**
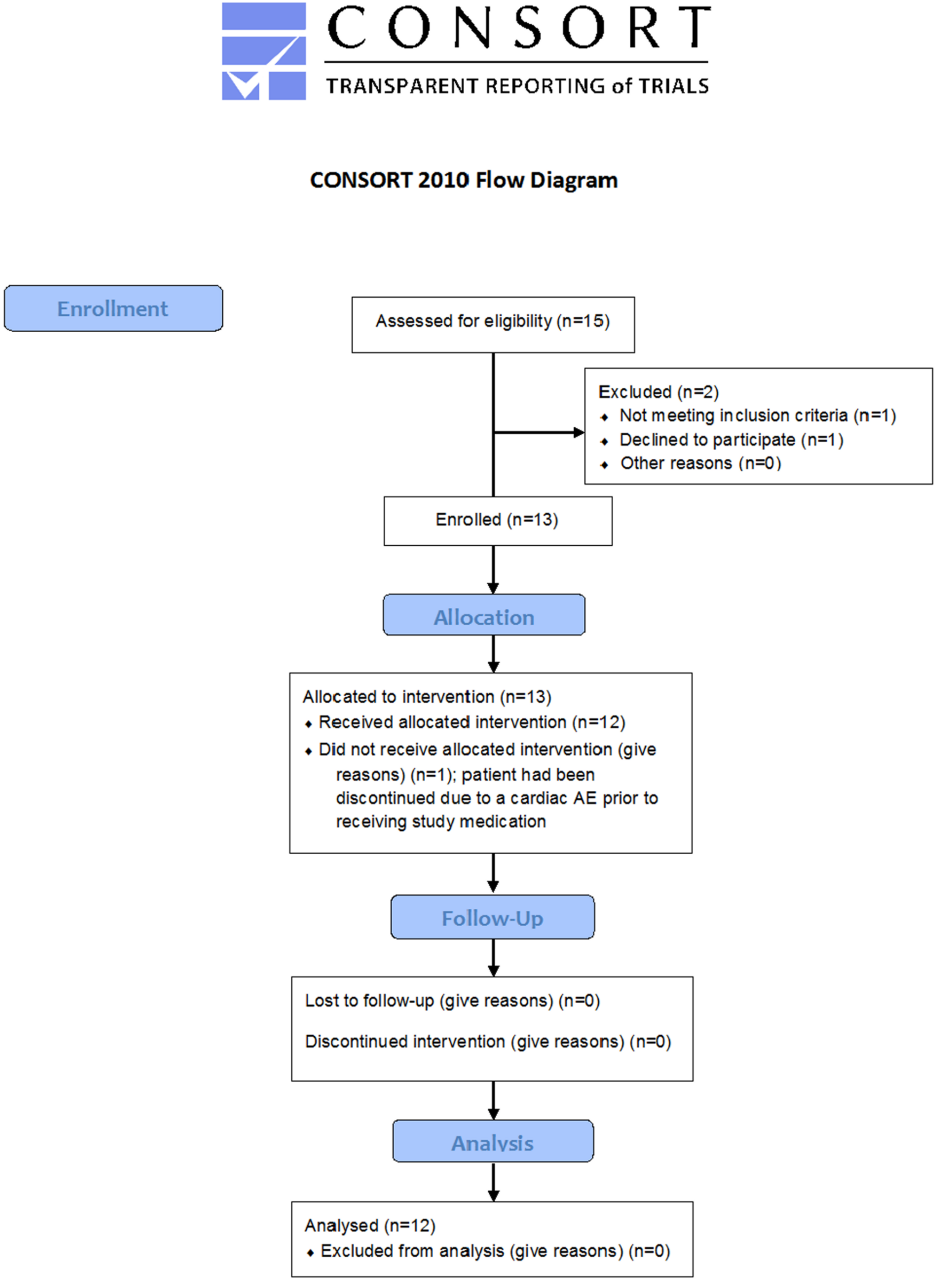
CONSORT flow diagram. Overview indicates the patient allocation.

A total of 15 patients were enrolled in this study between April 2015 and August 2016. Twelve patients were included in the efficacy and safety analysis (one subject had failed screening, one had withdrawn consent, and one had been discontinued due to a cardiac AE, all prior to receiving study treatment) (Fig 1). Three of the twelve POAG patients included had been diagnosed with normal tension glaucoma, one each for DL 2, 3 and 4. Mean time since diagnosis of POAG was 12.3 ± 11.2 years (Table 1). At the time-point of inclusion all patients had been scheduled to undergo trabeculectomy as per decision of their treating ophthalmologist. The mean preoperative IOP at this time-point was 27.3 mmHg +/- 12.6 mmHg (SD). Detailed demographics are depicted in Table 1. The average number of anti-glaucoma medications at the time of decision for surgery was 3.3 ± 0.78 (substances), with use of prostaglandins in 10, beta-blockers in 10, carbonic anhydrase inhibitors in 10, and adrenergic agonists in 10 patients, respectively (Table 2).

**Table 1:** Demographics.

	Dose Level 1	Dose Level 2	Dose Level 3	Dose Level 4	Total
	6.75 μg	22.5 μg	67.5 μg	225 μg	
	(0.3 μM)	(1 μM)	(3 μM)	(10 μM)	
	N = 3 (100%)	N = 3 (100%)	N = 3 (100%)	N = 3 (100%)	N = 12 (100%)
Age (years)					
Mean	65.0	63.0	66.7	70.0	66.2
Median	68.0	64.0	64.0	68.0	67.0
SD	± 13.75	± 16.52	± 4.62	± 5.29	± 10.01
Range	50–77	46–79	64–72	66–76	46–79
Gender					
male, number (%)	1 (33.3)	2 (66.7)	2 (66.7)	2 (66.7)	7 (58.3)
female, number (%)	2 (66.7)	1 (33.3)	1 (33.3)	1 (33.3)	5 (41.7)
Type of POAG					
high tension POAG	3 (100.0)	2 (66.6)	2 (66.6)	2 (66.6)	9 (75.0)
normal tension POAG	0 (0.0)	1 (33.3)	1 (33.3)	1 (33.3)	3 (25.0)
Involved eye					
OD	0 (0.0)	0 (0.0)	0 (0.0)	0 (0.0)	0 (0.0)
OS	2 (66.7)	0 (0.0)	0 (0.0)	0 (0.0)	2 (16.7)
OU	1 (33.3)	3 (100.0)	3 (100.0)	3 (100.0)	10 (83.3)
Time since diagnosis of POAG (years)					
Mean	20.0	9.3	16.3	3.3	12.3
Median	23.0	10.0	10.0	3.0	8.5
SD	±15.72	± 15.72	± 13.65	± 1.53	± 11.18
Range	3–34	7–11	7–32	2–5	2–34
IOP (mmHG) prior to trabeculectomy					
Mean	36.3	23.3	20.5	28.83	27.25
Median	27.0	23.0	23.0	26.0	23.8
SD	± 18.82	± 9.5	± 4.77	± 13.48	± 12.56

**Table 2:** Preoperative anti-glaucoma medication.

	Dose Level 1	Dose Level 2	Dose Level 3	Dose Level 4	Total
	6.75 μg	22.5 μg	67.5 μg	225 μg	
	(0.3 μM)	(1 μM)	(3 μM)	(10 μM)	
	N = 3 (100%)	N = 3 (100%)	N = 3 (100%)	N = 3 (100%)	N = 12 (100%)
Number of preoperative anti-glaucoma medication components^a^	3 (100) 10	3 (100) 12	3 (100) 10	3 (100) 8	12 (100) 40
Mean^b^	3.3	4.0	3.3	2.7	3.3
SD	1.15	0.00	0.58	0.58	0.78
Range^c^	2–4	4–4	3–4	2–3	2–4
Drug class^a^					
β-blocking agents	2 (66.7) 2	3 (100) 3	3 (100) 3	2 (66.7) 2	10 (83.3) 10
Carbonic anhydrase inhibitors	3 (100) 3	3 (100) 3	1 (33.3) 1	3 (100) 3	10 (83.3) 10
Prostaglandin analogues	2 (66.7) 2	3 (100) 3	3 (100) 3	2 (66.7) 2	10 (83.3) 10
Adrenergic agonists	3 (100) 3	3 (100) 3	3 (100) 3	1 (33.3) 1	10 (83.3) 10

^a^numbers indicate patients per dose level receiving medication (percentage of patients) total number of anti-glaucoma medication components/substances

^b^Mean: total number of preoperative anti-glaucoma medication components/number of patients receiving preoperative anti-glaucoma medication

^c^Range: lowest and highest number of preoperative anti-glaucoma medication components for patients in this dose group

### Safety results

All twelve patients treated with ISTH0036 completed the dose limiting toxicity monitoring period at Day 43 and the end of study assessments at 12 weeks follow-up. No DLT occurred. A total of 36 AEs was recorded during this observation period. None was declared to be related to ISTH0036 or the intravitreal injection procedure. 16 were declared to be related to the primary surgery and 20 as not related to investigational drug, to the intravitreal injection procedure itself or surgery. Two serious adverse events occurred during the clinical study in one patient (choroidal effusion, ocular hypertension) in dose cohort 1 (0.3 μM) with both events assessed as not related to ISTH0036 or the intravitreal injection procedure but certain and probably/likely related to primary surgery. Of the 16 clinical AEs declared to be related to primary surgery nine were Grade 1 or 2 eye disorders, three Grade 3 eye disorders, four Grade 1 or 2 events were classified as investigations. No Grade 4 AE occurred in the study. Two Grade 3 events occurred in DL 1 and one Grade 3 events in DL 2, none were observed with DL 3 and 4. See Table 3 for a detailed AE listing. As additional safety monitoring element, ERG evaluations were performed at screening and at 6 weeks follow-up. In total, 24 ERGs in 12 patients were recorded. Due to the small size of the study, only a descriptive patient-by-patient analysis was conducted and no quantitative evaluations were performed. No relevant safety findings were identified by local readers. This was confirmed by a post-hoc external central review.

**Table 3:** Adverse events by relationship, type and grade.

SOC	Adverse event (CTCAE Grade^b^)	Dose Level 1	Dose Level 2	Dose Level 3	Dose Level 4	Total
		6.75 μg	22.5 μg	67.5 μg	225 μg	
		(0.3 μM)	(1 μM)	(3 μM)	(10 μM)	
		N = 3	N = 3	N = 3	N = 3	N = 12
**Overall adverse events, n (%)**		7 (19.4)	12 (33.3)	11 (30.6)	6 (16.7)	36 (100)
**Related**^a^ **to ISH0036**		***None***	***None***	***None***	***None***	***None***
**Related**^a^ **to intravitreal injection**		***None***	***None***	***None***	***None***	***None***
**Related**^a^ **to primary surgery**		**3**	**7**	**4**	**2**	**16**
** Eye disorders:**		**3**	**6**	**1**	**2**	**12**
	*Corneal erosion*	*0*	*1*	*0*	*0*	*1*
	*Corneal epithelium defect*	*0*	*1*	*0*	*0*	*1*
	*Astigmatism*	*0*	*0*	*0*	*1 (Gr*.*2)*	*1 (Gr*.*2)*
	*Choroidal effusion*^c^	*1 (Gr*.*3)*	*0*	*0*	*0*	*1 (Gr*.*3)*
	*Conjunctival hemorrhage*	*0*	*0*	*0*	*1 (Gr*.*2)*	*1 (Gr*.*2)*
	*Conjunctival hyperaemia*	*0*	*1 (Gr*.*2)*	*0*	*0*	*1 (Gr*.*2)*
	*Corneal neovascularisation*	*0*	*0*	*1*	*0*	*1*
	*Corneal oedema*	*0*	*1*	*0*	*0*	*1*
	*Dry eye*	*0*	*1 (Gr*.*2)*	*0*	*0*	*1 (Gr*.*2)*
	*Eczema eyelids*	*1*	*0*	*0*	*0*	*1*
	*Ocular hypertension*^c^	*1 (Gr*.*3)*	*0*	*0*	*0*	*1 (Gr*.*3)*
	*Visual acuity reduced*	*0*	*1 (Gr*.*3)*	*0*	*0*	*1 (Gr*.*3)*
** Investigations:**		**0**	**1**	**3**	**0**	**4**
	*Intraocular pressure decreased*	*0*	*1*	*1 (Gr*.*2)*	*0*	*2 (1x Gr*.*2)*
	*Intraocular pressure increased*	*0*	*0*	*2 (Gr*.*2)*	*0*	*2 (Gr*.*2)*
**Unrelated**^a^		**4**	**5**	**7**	**4**	**20**
** Eye disorders:**		**1**	**2**	**3**	**2**	**8**
	*Corneal erosion*	*1*	*0*	*1 (Gr*.*2)*	*0*	*2 (1x Gr*. *2)*
	*Conjunctival oedema*	*0*	*0*	*1 (Gr*.*2)*	*0*	*1 (Gr*.*2)*
	*Corneal epithelium defect*	*0*	*1 (Gr*.*2)*	*0*	*0*	*1 (Gr*.*2)*
	*Erythema of eyelid*	*0*	*0*	*0*	*1 (Gr*.*2)*	*1 (Gr*.*2)*
	*Lacrimation increased*	*0*	*0*	*1*	*0*	*1*
	*Ocular hyperaemia*	*0*	*1 (Gr*.*2)*	*0*	*0*	*1 (Gr*.*2)*
	*Vision blurred*	*0*	*0*	*0*	*1*	*1*
** Infections and infestations:**		**1**	**2**	**2**	**1**	**6**
	*Nasopharyngitis*	*1*	*2*	*0*	*1 (Gr*.*2)*	*4 (1x Gr*.*2)*
	*Gingivitis*	*0*	*0*	*1*	*0*	*1*
	*Urinary tract infection*	*0*	*0*	*1*	*0*	*1*
** Investigations:**		**0**	**1**	**0**	**1**	**2**
	*Intraocular pressure increased*^d^	*0*	*1 (Gr*.*2)*	*0*	*0*	*1 (Gr*.*2)*
	*Vital dye staining cornea present*	*0*	*0*	*0*	*1 (Gr*.*2)*	*1 (Gr*.*2)*
** Immune system disorders:**	*Drug hypersensitivity*	**1**	**0**	**0**	**0**	**1**
** Metabolism and nutrition disorders:**	*Gout*	**0**	**0**	**1** (Gr.2)	**0**	**1** (Gr.2)
** Musculoskeletal and connective tissue disorders:**	*Muscle spasms*	**1**	**0**	**0**	**0**	**1**
** Vascular disorders:**	*Hypertension*	**0**	**0**	**1**	**0**	**1**

^a^Reasonable possibility (certain, probable/likely, possible); no reasonable possiblity (unlikely related or unrelated to ISTH0036, intravitreal injection and primary surgery)

^b^AEs with intencity CTCAE Grade ≥ 2 are indicated in brackets; all other AEs are CTCAE Grade 1

^c^Serious adverse event

^d^Non-study eye

### Efficacy data

To determine potential preliminary clinical efficacy of ISTH0036 postoperative intraocular pressure course, number of interventions post TE, bleb morphology, best corrected visual acuity, visual field, slit lamp biomicroscopy, and optic disc status (Heidelberg Retinograph II (HRTII) and photograph of the optic disc) were recorded as secondary endpoints at defined time-points.

**Fig 2:**
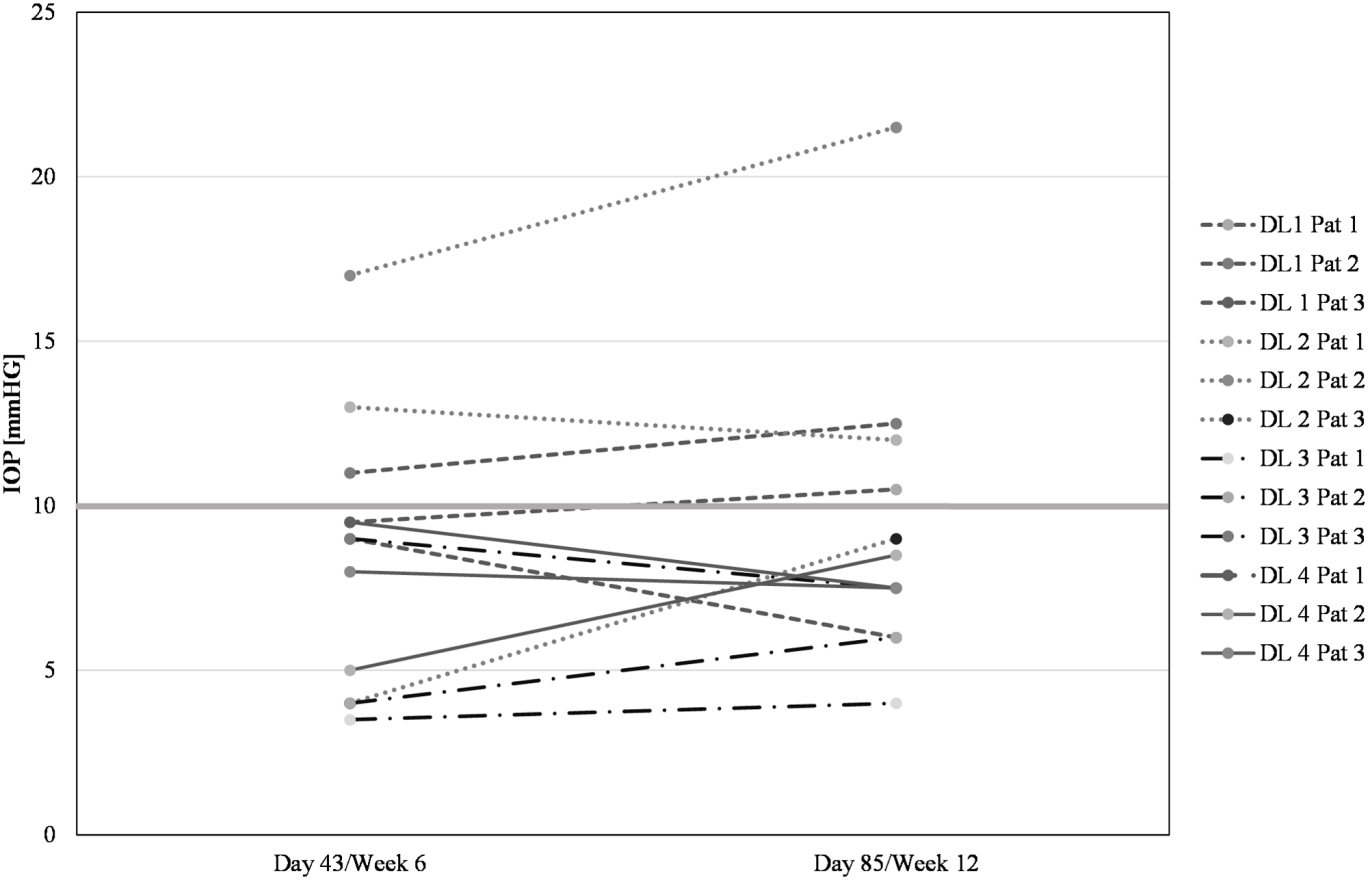
Postoperative intraocular pressure on Day 43 and Day 85 per patient. Bar indicates 10 mmHg IOP level threshold not exceeded by DL 3 and DL 4 patients.

**Fig 3:**
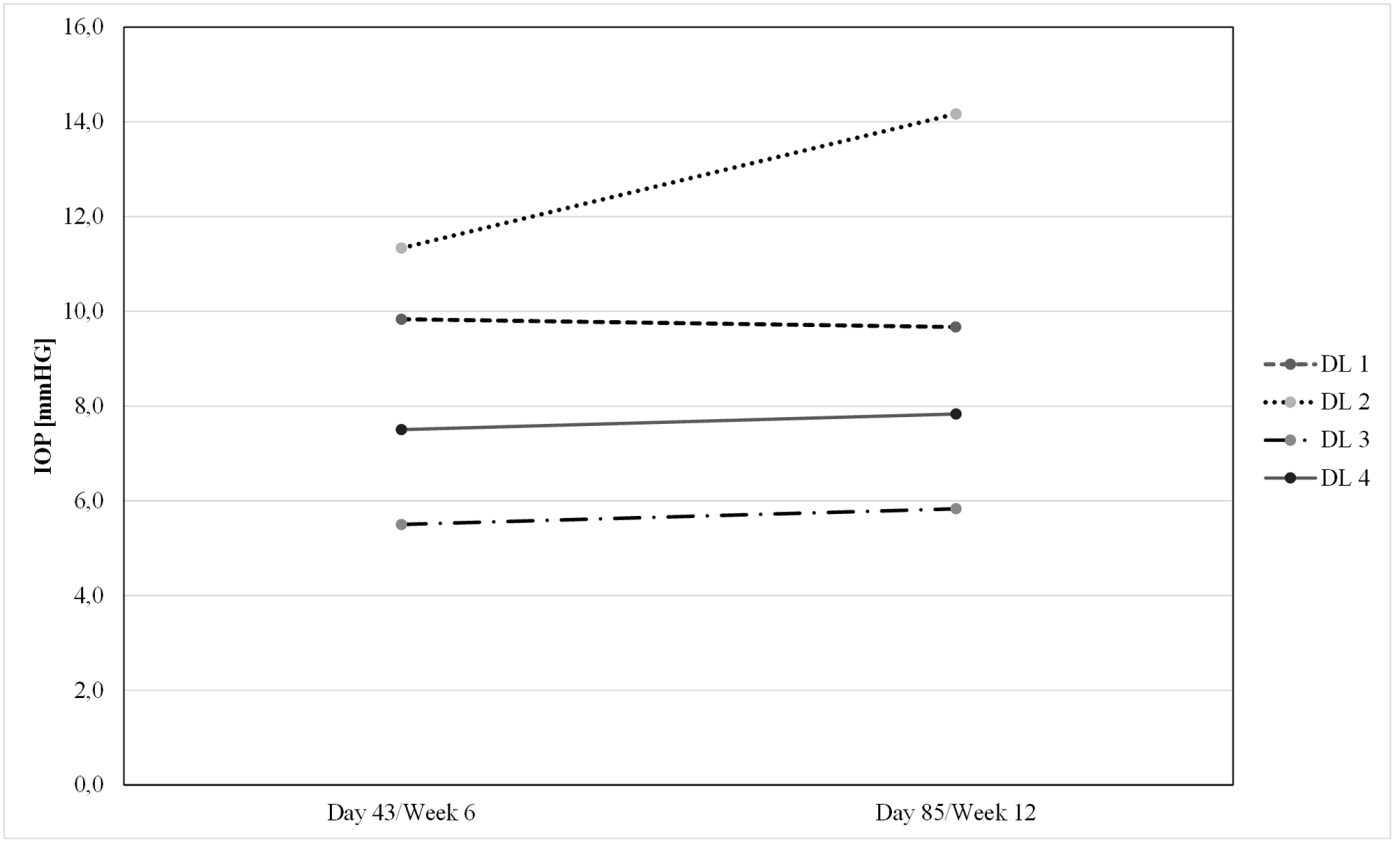
Mean intraocular pressure on Day 43 and Day 85 per dose level.

The mean preoperative IOP of all subjects at the time of decision for surgery was 27.3 mmHg ± 12.6 mmHg (SD). After trabeculectomy with standard administration of MMC and single intravitreal injection of ISTH0036, mean postoperative IOP values (± SD) for dose level 1, 2, 3 and 4 were at 6 weeks (Day 43) 9.8 mmHg (± 1.0 mmHg), 11.3 mmHg (± 6.7 mmHg), 5.5 mmHg (± 3.0 mmHg), and 7.5 mmHg (± 2.3 mmHg); and at 12 weeks (Day 85) 9.7 mmHg (± 3.3 mmHg), 14.2 mmHg (± 6.5 mmHg), 5.8 mmHg (± 1.8 mmHg) and 7.8 mmHg (± 0.6 mmHg), respectively (see Fig 2 for individual postoperative IOP course and Fig 3 for mean IOP course per dose level). In contrast to DL 1 and 2, IOP values for DL 3 and 4 consistently remained below 10 mmHg throughout the postoperative observation period (up to Day 85). These results are potentially indicative of a dose-response trend.

For most other efficacy parameters no clear trends or significant alterations were observed: number of interventions post trabeculectomy (overview see Table 4), visual field, slit lamp biomicroscopy, and optic disc status did not show any trends in any direction, in line with the duration of the observation period and the small patient number typical for a phase I study. For bleb filtering and bleb morphology there was overall a trend for increase in bleb score over time (except for DL 2; see Table 5 for details). In line with prior observations for trabeculectomy best corrected visual acuity (BCVA) worsened moderately in all patients at all dose levels for the observation period (Table 6), as described in other trabeculectomy outcome analyses, too [27].

**Table 4:** Postoperative interventions.

Intervention	Dose Level 1	Dose Level 2	Dose Level 3	Dose Level 4	Total
	6.75 μg	22.5 μg	67.5 μg	225 μg	
	(0.3 μM)	(1 μM)	(3 μM)	(10 μM)	
	N = 3 (100%)	N = 3 (100%)	N = 3 (100%)	N = 3 (100%)	N = 12 (100%)
Number	3 (100) 8	3 (100) 8	3 (100) 14	2 (66.7) 10	11 (91.7) 40
Mean	2.7	2.7	4.7	3.3	3.3
SD	± 2.08	± 2.08	± 2.31	± 4.16	± 2.53
Range	1–5	1–5	2–6	0–8	0–8
Type					
5-FU	5 (62.5)	4 (50.0)	5 (35.7)	5 (50.0)	19 (47.5)
Suturolysis	1 (12.5)	2 (25.0)	4 (28.6)	4 (40.0)	11 (27.5)
Bulbus massage	1 (12.5)	0	2 (14.3)	1 (10.0)	4 (10.0)
Other^a^	1 (12.5)	2 (25.0)	3 (21.4)	0	6 (15.0)

^a^Other: 1x addition of nylon sutures, 1x therapeutic lens and other therapeutics 3x glucocorticoid or 1x cycloplegic agents.

**Table 5:** Bleb filtering and morphology evaluation (Wuerzburg bleb classification Score).

	Wuerzburg Bleb Score	Wuerzburg Bleb Score	Wuerzburg Bleb Score	Wuerzburg Bleb Score
	Dose Level 1	Dose Level 2	Dose Level 3	Dose Level 4
	6.75 μg (0.3 μM)	22.5 μg (1 μM)	67.5 μg (3 μM)	225 μg (10 μM)
	N = 3	N = 3	N = 3	N = 3
Day 3				
Mean	7.3	8.0	4.0	7.0
SD	± 2.52	± 2.65	± 0.00	± 2.00
Range	5–10	5–10	4–4	5–9
Day 43				
Mean	10.0	8.7	9.3	7.7
SD	± 0.00	± 1.53	± 2.08	± 3.21
Range	10–10	7–10	7–11	4–10
Day 85				
Mean	11.3	7.0	11.0	9.7
SD	± 0.58	± 1.00	± 0.00	± 1.53
Range	11–12	6–8	11–11	8–11

**Table 6:** Best corrected visual acuity (logMAR Score).

	logMAR Score	logMAR Score	logMAR Score	logMAR Score
	Dose Level 1	Dose Level 2	Dose Level 3	Dose Level 4
	6.75 μg (0.3 μM)	22.5 μg (1 μM)	67.5 μg (3 μM)	225 μg (10 μM)
	N = 3 (100%)	N = 3 (100%)	N = 3 (100%)	N = 3 (100%)
Screening				
Mean	0.40	0.21	0.01	0.09
SD	± 0.31	± 0.21	± 0.12	± 0.25
Baseline				
Mean	0.41	0.17	-0.03	0.14
SD	± 0.30	± 0.15	± 0.14	± 0.22
Day 3				
Mean	0.72	0.26	0.25	0.55
SD	± 0.16	± 0.00	± 0.13	± 0.55
Day 43				
Mean	0.58	0.41	0.23	0.28
SD	± 0.35	± 0.40	± 0.11	± 0.07
Day 85				
Mean	0.51	0.27	0.12	0.27
SD	± 0.36	± 0.28	± 0.16	± 0.13±

## Discussion

Glaucoma continues to be a threat to visual function for millions of patients worldwide. Despite multiple medical treatment options, in the US alone more than 120.000 patients have turned bilaterally blind from glaucoma, representing 1/10 of all cases [27]. So far, the pathophysiology and underlying molecular mechanisms of glaucoma have not been fully understood [28]. However, IOP elevation plays a critical role in the majority of patients, and lowering of IOP can retard or even arrest progression of glaucoma. Retinal ganglion cell death appears to be initiated and impacted by several pathophysiologic mechanisms.

One important mechanism is tissue and extracellular matrix remodeling which may lead to trabecular meshwork alterations and subsequently IOP rise and tissue structure alterations of the optic nerve head, all resulting in direct and indirect optic nerve head damage. TGF-β2 has been recently identified as a key driver in those mechanisms and is now targeted for drug development [29]. In this context it is suggested that targeting TGF-β2 may (1) provide potent anti-fibrosis/anti-scarring activity in trabeculectomy (2) mediate effective neuroprotection by blocking extracellular matrix remodeling, and (3) prevent trabecular meshwork alteration, (with 2) and (3) both being central to glaucoma disease pathophysiology. Consequently, TGF-ß2 has become a prime target in glaucoma. Noteworthy, TGF-β2 has also been linked to other key ophthalmic diseases such as wet and dry age-related macular degeneration (AMD), diabetic retinopathy, proliferative vitreoretinopathy and corneal diseases, being involved in tissue remodeling and fibrotic processes [30].

For advanced-stage glaucoma it is therefore highly desirable to explore novel treatment options to achieve more optimal IOP control, and protect visual field more efficiently. ISTH0036 could play here an important role, by not only providing improved postoperative IOP control but also by preventing further alteration of the trabecular meshwork and the direct pathologic impact of elevated TGF-β2 levels on the optic nerve head [11].

In this first clinical exploration ISTH0036 demonstrated to be safe when administered in patients undergoing trabeculectomy with MMC. No adverse event observed was linked to ISTH0036 and safety evaluations such as ERG did not indicate any toxicity by the compound. Regarding efficacy, a dose-response trend regarding IOP could be observed for the two highest doses evaluated. Remarkably, none of the six patients treated at these dose levels exceeded an IOP of 10 mmHg in the observation period (12 weeks), maintaining early postoperative IOP in ranges shown to result in more favorable outcomes for patients [31]. No hypotony of clinical concern was observed. Despite the limited size of this phase I study and not being designed to demonstrate efficacy on statistically significant level this finding is suggestive of a potentially beneficial therapeutic effect.

An earlier exploration of anti-TGF-β treatment based upon an antibody (CAT-152) had failed to demonstrate a clinical benefit [32]. This failure appeared to be due to several limitations: The dosing strategy for CAT-152 appeared suboptimal (administration just between Day -1 and Day 8 although the scarring process is expected to last for many months, a comparatively short half-life and pharmacodynamic effect of the compound, with limited PK/PD explorations available. Furthermore, the subconjunctival administration might also have contributed to the lack of efficacy. ISTH0036 may provide a different option here, with long tissue half-life and pharmacodynamic activity persisting for up to 8 weeks in preclinical in vivo models. Therefore, repeat dosing up to one year is planned for phase II studies to maintain an anti-fibrotic effect for clinically relevant time-periods.

Despite various surgical options, trabeculectomy remains a standard surgical intervention by which aqueous humor is drained from the anterior chamber to the subconjunctival space forming a filtration bleb. Alternative methods, such as microinvasive surgical techniques, have been developed but did not surpass the results achieved with trabeculectomy nor replace it [33]. Excessive wound healing in the subconjunctival space is the major threat for long-term success with glaucoma filtration surgery (and the alternative surgical interventions) and is the most frequent cause for failure of TE [5, 34]. While wound healing in general is a physiologic and positive phenomenon, in TE wound healing resulting in excessive scarring and closure of the surgically opened drainage canal and filtering bleb represents a major threat to success. Consequently, to suppress excessive scarring various agents have been explored in past decades in randomized trials, among them anti-metabolites such as 5-FU and MMC.

At present, solely MMC has been approved as an anti-scarring agent in trabeculectomy (US, only), but is frequently used on an off-label basis in other regions of the world. 5-FU appears to be overall significantly less active than MMC [35, 36]. In addition, corticosteroids are routinely used as postoperative anti-inflammatory agent with some, though quite limited anti-fibrotic activity. MMC has been explored in numerous studies in the past decades at different concentrations and exposure durations. Variability in study populations, dose/exposures explored and assessment time-point and endpoint variability do not allow for direct comparisons across most studies and make meta-analyses quite challenging. Yet, various clinical data support that MMC is effective in achieving IOP lowering versus (vs.) placebo when administered during trabeculectomy. In five randomized and prospective studies that were conducted between 1995 and 2015 [6, 37–40], MMC use resulted in mean IOP values of 10.6/11.0 mmHg at 3 months and 9.9/12.2/12.8/13.7/13.7 mmHg at 12 months. Various large retrospective studies [41] report similar IOP ranges achieved with MMC use post TE. In a meta-analysis Fendi et al. [42] analyzed 5 randomized, controlled trials (RCT) that compared MMC with 5-FU in TE. MMC use was associated with a statistically significantly lower mean IOP level following TE than 5-FU, with a mean IOP of 11.25 mmHg vs. 13.58 mmHg with 5-FU (p < 0.001). Three RCTs within this meta-analysis evaluated the qualified surgical success rates defined as IOP < 18 mmHg. The MMC group showed in a pooled analysis a higher qualified success rate (130/156 [83.3%]) than the 5-FU group (111/148 [75.0%]; p = 0.04). In conclusion, prospective as well as retrospective studies and meta-analyses have shown that MMC as adjuvant results in lower IOP as compared to placebo or 5-FU. Achieved IOP improvement vs. placebo or other controls ranged depending on study and conditions/population from roughly 2.0 to over 5.0 mmHg in the first year.

MMC is effective in maintaining a comparatively lower IOP post trabeculectomy by an anti-scarring effect vs. other agents. However, this effect comes at a price. The Center for Drug Evaluation review of MMC (Mitosol^™^) lists as most frequent adverse reactions to topical MMC use in trabeculectomy hypotony, choroidal detachment, shallow anterior chamber, hyphema, corneal endothelial defects, and cataract progression. Frequencies range from 0–3% but also from 30–50% within different studies. This may depend on study design (retrospective versus prospective) but also on the adverse event reporting regulations applied [http://www.accessdata.fda.gov/drugsatfda_docs/nda/2012/022572Orig1s000MedR.pdf].

MMC does not block glaucoma-specific pathophysiologic processes nor does it prevent glaucoma progression apart from its anti-scarring effect after trabeculectomy. The fact that individuals still progress to blindness despite availability of effective surgical interventions and the frequency of postoperative IOP rises underlines the need for further improvement with effective agents targeting the underlying pathology of glaucoma.

Due to the need for improved IOP control and the suboptimal AE profile of MMC alternatives have been explored. Here in past years a main focus was on anti-vascular endothelial growth factor (VEGF) treatments, such as bevacizumab, ranibizumab, aflibercept. It is debatable whether the scarring process is mainly dependent upon vascularization, but wound healing disturbances observed with systemic use of anti-VEGF agents in cancer patients and molecular evidence for the role of VEGF in this regard [43] support a distinct role in the wound healing process. Yet, although some early trials suggested that anti-VEGF agents may have some effect against scarring and bleb failure, other reports fail to demonstrate this effect. A recent large meta-analysis of 9 studies with 349 patients came to the conclusion that antimetabolites are more effective in IOP lowering than anti-VEGF approaches, and that combining MMC with anti-VEGF compounds does not improve in a statistically significant way the complete or qualified success rate of trabeculectomy [44]. In the trabeculectomy setting some effects on IOP appear to be achievable on top of MMC, but again no improved success rates could be demonstrated so far [45]. Consequently, at present anti-VEGF treatment is not seen as a clinical standard in the trabeculectomy setting for POAG.

Recent scientific analyses have demonstrated that it is highly desirable to stabilize the early postoperative IOP in ranges below 10 mmHg, in some cases even below 8 mmHg [31]. While by the use of MMC alone such IOP ranges are rarely reached, achieving such IOP is highly desirable as this is indicative of long-term success of trabeculectomy. It heralds better clinical outcome for the patient, both in POAG and in closed-angle glaucoma [31, 46],.

A randomized, prospective trial will be needed to clarify if ISTH0036 may be efficacious in combination with MMC or even may be sufficiently efficacious as monotherapy alone. Therapeutic target would be to achieve consistent IOP ranges of 6–10 mmHg for patients post trabeculectomy and to provide significantly improved IOP control over MMC alone. Naturally, hypotony rates need to be monitored carefully in phase II to determine whether the combination with MMC may not be too potent and may result in too low IOPs for some patients. In addition, in monotherapy or combination, additional potentially beneficial effects such as neuroprotection and trabecular meshwork protection by preventing further extracellular matrix remodeling of the optic nerve head area and the trabecular meshwork may contribute further to a potential beneficial effect.

Noteworthy, TGF-β has not only been shown to be involved in glaucoma pathophysiology, but in recent years has also been linked to various other major ophthalmic diseases such as wet [47] and dry [30] AMD, diabetic retinopathy [48], proliferative vitreoretinopathy [49, 50], secondary cataract [51] and corneal disease [52]. With its anti-fibrotic and antiangiogenic effects ISTH0036 currently also undergoes preclinical evaluation in other disease models than glaucoma to consider expansion of development into these disease entities.

In summary, this first-in-human study of ISTH0036 demonstrates that selective suppression of TGF-β2 with single doses of this LNA-based antisense oligonucleotide is safe and potentially clinically efficacious. Signs of a potentially meaningful clinical activity have been observed in terms of a dose-response trend and encouraging IOP control < 10 mmHg for the first 12 weeks for all patients treated at dose levels 3 and 4 (67.5 μg or 225 μg dose). In the light of its potent anti-fibrotic effect, but also the suspected capability to block major glaucoma pathophysiology mechanisms (epithelial-to-mesenchymal transition of the trabecular meshwork, extracellular matrix remodeling affecting the optic nerve head, other mechanisms) ISTH0036 warrants further clinical evaluation in glaucoma filtration surgery. Due to the potent anti-fibrotic effect and the additionally observed potent antiangiogenic effect other ophthalmic diseases that are linked to TGF-β2 (wet AMD, dry AMD, diabetic retinopathy, proliferative vitreoretinopathy) may also be explored for ISTH0036 development.

## Supporting information

S1 File Clinical study protocol, version 3.0, 05 Sep 2016. (PDF)

S2 File TREND statement checklist. (PDF)

S3 File Intraocular pressure prior to surgery and on Day 43 and Day 85 by patient. (PDF)

S1 Fig Intraocular pressure prior to surgery and on Day 43 and Day 85 by patient. (TIF)
