# Supplementary material for: First-in-human phase I study of ISTH0036, an antisense oligonucleotide selectively targeting transforming growth factor beta 2 (TGF-β2), in subjects with open-angle glaucoma undergoing glaucoma filtration surgery
Source: PLoS One. 2017 Nov 30;12(11):e0188899. doi: 10.1371/journal.pone.0188899 (PMC5708654; doi:10.1371/journal.pone.0188899)
Supplement: S1 File — (PDF) [file pone.0188899.s001.pdf]

Clinical Study Protocol

**Protocol Title:**	A phase I, open-label, dose-escalation study to investigate the safety of ISTH0036, a ‘next generation’ TGF-β2-selective antisense oligonucleotide, in subjects with primary open-angle glaucoma undergoing trabeculectomy
**Study Number**:	ISTH-01-111
**EudraCT-Number:**	2014-004985-74
**Short Title:**	ISTH0036 in trabeculectomy
**Version Number/Date:**	**FINAL 3.0**, 05 Sep 2016
**Amendment Number/Date:**	**Amendment No. 2, Version 1.0,** 05 Sep 2016

Sponsor:
Dr. Philippe Calais, CEO
Isarna Therapeutics GmbH
Leopoldstr. 254-256, 80807 Munich | Germany
www.isarna-therapeutics.com
Phone: +49-89-890 831-0, Fax: +49-89-890 831-491
p.calais@isarna-therapeutics.com

Coordinating Investigator:
Prof. Dr. med. Norbert Pfeiffer
Department of Ophthalmology, University Medical Center
Johannes Gutenberg-University Mainz
Building 101
Langenbeckstr. 1
55131 Mainz
Germany
Phone: +49-6131-17-7085, Fax: +49-6131-17-5509
norbert.pfeiffer@unimedizin-mainz.de

The information herein is the property of Isarna Therapeutics GmbH and may not be reproduced, published or disclosed to others without written authorization of Isarna Therapeutics GmbH.

Sponsor Signature Page

Sponsor's Chief Executive Officer

Dr. Philippe Calais

Date: SEP 8, 2016 Signature: ______________________

Sponsor's Head Clinical Research and Development

Prof. Dr. med. Eugen Leo

Date: Sep 8, 2016 Signature: ______________________

Sponsor's Head Clinical Operations

Dr. Petra Fettes

Date: 05. Sept. 2016 Signature: ______________________

l

Coordinating Investigator for Germany Signature Page (LKP according to German Drug Law § 40 (1) No. 5)

Prof. Dr. med. Norbert Pfeiffer

Date: 08.09.16 Signature: [signature]

Investigator Signature Page

Protocol No.: ISTH-01-111

Protocol Title: A phase I, open-label, dose-escalation study to investigate the safety of ISTH0036, a ‘next generation’ TGF-β2-selective antisense oligonucleotide, in subjects with primary open-angle glaucoma undergoing trabeculectomy

Short Title: ISTH0036 in trabeculectomy

Study Site No.: *< Enter site no >*

I have read and understood the protocol and agree to conduct the study in compliance with the protocol, Good Clinical Practice (GCP) and all applicable national or regional regulations/guidelines. In addition, I will conduct the study in accordance with the ethical principles of the Declaration of Helsinki.

I agree to assume responsibility for the proper conduct of the study at this site and I will ensure that all persons assisting in the study under my supervision are adequately informed about the protocol/amendments, the investigational product and their study related duties and functions as described in the protocol.

I will not implement any deviation from, or changes to the protocol without agreement from the sponsor and prior submission to and written approval from (where required) the responsible regulatory authorities and institutional review board (IRB) / independent ethics committee (IEC) of an amendment, except when necessary to eliminate an immediate hazard to the study subjects.

I understand that the study may be terminated or enrollment may be suspended at any time by Isarna Therapeutics, with or without cause, or by myself if it becomes necessary to protect the best interest of the study subjects.

< Enter name of investigator >

< Address >

< Phone >

< Fax >

< E-mail address >

Date: ____________ Signature: _________________________

List of Terms and Abbreviations

Term or Abbreviation	Description
5-FU	5-fluorouracil
ß-HCG	beta human chorionic gonadotropine
ADR	adverse drug reaction
AE	adverse event
ALAT	alanine aminotransferase
ASO	antisense oligonucleotide
ASAT	aspartate aminotransferase
BMP	bone morphogenetic protein
CA	Competent Authority
CRC	Cohort Review Committee
CRF	Case Report Form
CRO	clinical Research Organization
CSLO	Confocal scanning laser ophthalmoscopy
CTCAE	Common Terminology Criteria for Adverse Events
CTGF	connective tissue growth factor
DLT	dose limiting toxicity
DNA	deoxyribonucleic acid
DSUR	Development Safety Update Report
eCRF	electronic Case Report Form
EC	Ethics Committee
ECM	extracellular matrix
EDC	electronic data capture
ETDRS	early Treatment Diabetic Retinopathy Study
EOS	end of study
ERG	electroretinogram
FAS	full analysis set
FU	follow-up
GCP	Good Clinical Practice
GGT	γ-glutamyltransferase
GLP	Good Laboratory Practice
HIV	human immunodeficiency virus
HRT	Heidelberg retina tomograph
ICF	Informed Consent Form
ICH	International Conference on Harmonization
IEC	independent ethics committee
IMP	Investigational Medicinal Product
INR	International normalized ratio of prothrombin time
IOL	intra ocular lens
IOP	intraocular pressure
IRB	Institutional Review Board
IVT	intravitreal
LDH	lactate dehydrogenase
LNA	locked nucleic acid

LogMAR	Logarithm of the minimum angle of resolution
LTBP2	latent TGF-β binding protein 2
MedDRA	Medical Dictionary for Regulatory Activities
MMC	mitomycin C
MMP	matrix metalloprotease
mRNA	messenger RNA
NCI	National Cancer Institute
NDO	no drug-related adverse observation
NOAEL	no adverse effect level
PAI-1	plasminogen activator inhibitor 1
POAG	primary open angle glaucoma
PPS	per-protocol analysis set
RBC	red blood cell count
RNA	ribonucleic acid
SAE	serious adverse event
SAF	safety analysis set
SAP	Statistical Analysis Plan
SOC	system organ class
SOP	standard operating procedure
SUSAR	suspected unexpected serious adverse reaction
TEAE	treatment emergent adverse event
TGF-β	transforming growth factor-β
TLF	tables, listings, and figures
TMF	trial master file
VEGF	vascular endothelial growth factor
WBC	white blood cell count

List of Contacts

SERVICE PROVIDER	
**Clinical Research Organization (Monitoring, Data Management and Statistics)**	
SynteractHCR Deutschland GmbH<br>Albrechtstrasse 14<br>80636 Munich, Germany	
**Pharmacovigilance**	
spm² - safety projects & more GmbH<br>Aurum 05<br>Goldbeckstr. 5<br>69493 Hirschberg a. d. Bergstraße, Germany	phone: +49 (0)6201 84 64 0 - 0<br>fax: +49 (0)621 570 - 5971<br>email: Isarna-PhV@spm2-safety.com
**Drug Supplies Management**	
Theorem Clinical Research GmbH<br>Am Kronberger Hang 3<br>65824 Schwalbach, Germany	

TABLE OF CONTENTS

List of Terms and Abbreviations 5
List of Contacts 7
List of Tables 9
2.6.1 Benefits 19
2.6.2 Risks 20
4.1.1 Primary Endpoint(s) 21
4.1.2 Secondary Endpoint(s) 21
6.1.1 Formulation, Packaging and Labeling 23
6.1.2 Storage and Stability 24
6.1.3 Preparation 24
6.1.4 Drug Accountability 26
6.1.5 Destruction of IMP 26
7.1.1 Treatment Assignment 26
7.1.2 Administration 26
8.3.1 Visual acuity 30
8.3.2 Visual field 30
8.3.3 Slit-lamp biomicroscopy 30
8.3.4 Bleb filtering evaluation 30
8.3.5 Intraocular Pressure Measurements 31
8.3.6 Heidelberg Retina Tomograph (HRTII) 31
8.3.7 Optic disc photographs 31
9.2.1 Visit 2 (hospitalization) 32
9.4.1 In Case of Adverse Events 33
9.4.2 In Case of Signs and Symptoms of Disease Progression 34
9.6.1 Post-Study Visit 6 months – PSFU 1 34
9.6.2 Post-Study Visit 12 months – PSFU 2 34
9.6.3 Post-Study Follow-Up (PSFU 3) 34
11.1.1 Adverse Events 35
11.1.2 Serious Adverse Event 36
11.2.1 Observation period 37
11.2.2 Assessment of Seriousness 37
11.2.3 Assessment of Severity 37
11.2.4 Assessment of Causality 37
11.4.1 Reporting of Serious Adverse Events 39
11.4.2 Other Reportable Information 40
11.5.1 SUSAR Reporting 40

11.5.2 Developmental Safety Update Report (DSUR) ... 40
12.1.1 Statistical Hypothesis ... 41
12.1.2 Level of Significance, Multiple Comparisons and Multiplicity ... 41
12.1.3 Determination of Sample Size ... 41
12.2.1 Demographic Data and Other Baseline Characteristics ... 41
12.2.2 Planned Analysis for Primary Endpoint(s) ... 41
12.2.3 Planned Analysis for Secondary Endpoint(s) ... 42
12.2.4 Further variables ... 42
Appendix 1 Study Flow Charts ... 54
Appendix 2 Cohort Review Committee Process ... 56
Appendix 3 Slit-lamp biomicroscopy evaluations ... 58
Appendix 4 Trabeculectomy Standard-operating-procedure (SOP) ... 61
Appendix 5 Intravitreal Injection SOP ... 63
Appendix 6 Intra ocular pressure measurement using the Goldmann tonometer SOP ... 64
Appendix 7 Heidelberg Retina Tomograph II (HRTII) Ophthalmoscopy Interpretation (German and English) ... 65
Appendix 8 NCI CTCAE classification ... 69

List of Tables

Table 1: Summary of preclinical toxicity assessments of the 4-week toxicology study in Dutch-Belted rabbit .. 18
Table 2: Secondary endpoints and corresponding methods of assessment ... 21
Table 3: Planned dose levels and corresponding reconstitution ... 25
Table 4: Wuerzburg Bleb Classification Score ... 30
Table 5: Mapping of causality categories to binary causality assessment ... 38
Table 6: Study Flow Chart ... 54

1 STUDY SYNOPSIS

Title of Study

A phase I, open-label, dose-escalation study to investigate the safety of ISTH0036, a ‘next generation’ TGF-β2-selective antisense oligonucleotide, in subjects with primary open-angle glaucoma undergoing trabeculectomy

Sponsor

Isarna Therapeutics GmbH, Leopoldstrasse 254-256, 80807 Munich, Germany

Coordinating Investigator (LKP according to German Drug Law § 40 (1) No. 5)

Prof. Dr. med. Norbert Pfeiffer, Department of Ophthalmology, University Medical Center Johannes Gutenberg-University Mainz, Building 101, Langenbeckstr. 1, 55131 Mainz, Germany

Study Site(s)

Multicenter trial, 3 sites

Countries

Germany

Phase of Development

I

Study Rationale

The cytokine human transforming growth factor beta (TGF-β) is known to play a key role in various ophthalmic diseases. In glaucoma, increased levels of TGF-β2 present in the eye have been linked to trabecular meshwork transformation, increased intraocular pressure and direct optic nerve damage.

In advanced stage glaucoma trabeculectomy is the standard surgical intervention to reduce intraocular pressure in subjects not responding sufficiently anymore to pressure-lowering medications. Yet, scarring of the surgically opened canal (“bleb-closure”) often abolishes the effect of trabeculectomy (despite the intraoperative use of Mitomycin C to prevent this) and the surgical intervention itself does not block core glaucoma pathophysiologic processes. Importantly, TGF-β2 has been described to play a distinct role in the fibrotic process of bleb closure.

Consequently, blocking the effect of TGF-β2 by a selective antisense oligonucleotide (ASO) in the context of trabeculectomy appears to be an attractive potential therapeutic concept to (1) prevent bleb closure, (2) block any key pathophysiology of TGF-β2 in glaucoma progression (trabecular meshwork transformation with IOP-rise and optic nerve damage).

ISTH0036 is a ‘next generation’ antisense oligodeoxynucleotide based on locked-nucleic acid technology (LNA), selectively targeting TGF-β2, with favorable tissue penetration and tissue half-life. By forming complexes with TGF-β2 isoform mRNA, ISTH0036 blocks TGF-β2 protein production and results in degradation of the respective TGF-β2 isoform mRNA. Preclinical data have shown that TGF-β2 ASO has potent glaucoma progression preventing activity in various in vitro and in vivo preclinical models. Preclinical toxicology studies have shown that treatment with ISTH0036 is safe and overall well tolerated.

In summary, exploration of the ISTH0036 in the trabeculectomy setting appears justified and of high medical interest.

Objectives

Primary objective

- To determine the safety and tolerability of ISTH0036

Secondary objective

- To determine preliminary clinical efficacy of ISTH0036

Study Endpoints

Primary endpoint

- Type and frequency of adverse events

Secondary endpoint(s)

The following parameters will be assessed at different time points and compared to baseline. On ISTH0036 treatment days, all examinations will be done pre-dose.

- Intraocular pressure (Damiano *et al.*)
- Number of interventions post trabeculectomy
- Bleb filtering and bleb morphology
- Visual acuity
- Visual field
- Slit lamp biomicroscopy
- Optic disc status (Heidelberg Retinograph II (HRTII) and photograph of the optic disc)

Study Design

The study is a dose escalation study to explore the safety and tolerability of single doses of ISTH0036. Patients will be enrolled in cohorts of three patients to evaluate each dose level administered. Up to 4 different dose levels of ISTH0036 will be evaluated. The study medication will be administered as an intravitreal injection on Day 1 (during surgery, at the end of the trabeculectomy surgical procedure, after topical MMC administration). There must be a minimum interval of one week between the dosing of the first and all subsequent patients in each cohort. Dose escalation will occur when data from 3 subjects who have completed the dose limiting toxicity (DLT) period of 42 days is available and all available safety data have been reviewed by the Cohort Review Committee (CRC) and no DLT has been observed.

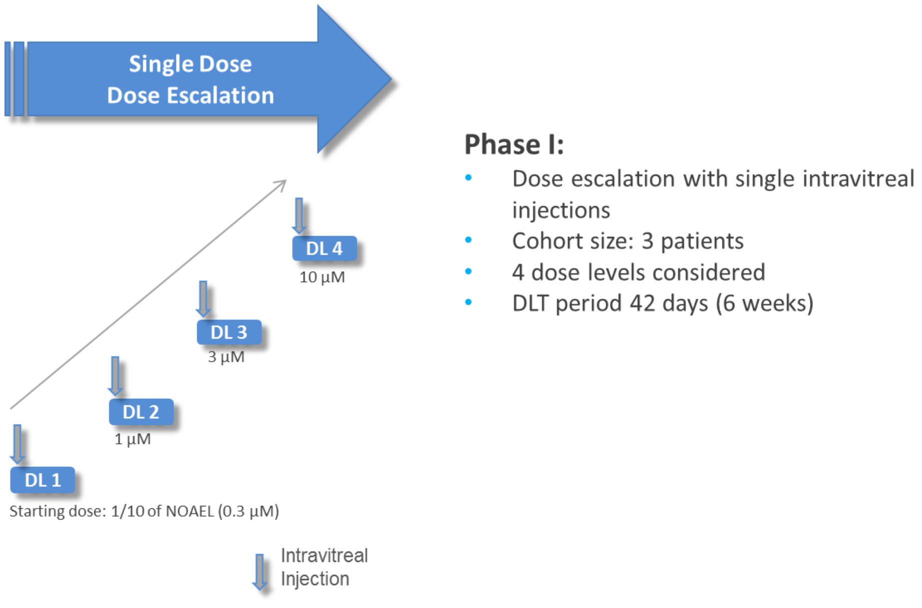

Number of Subjects

Approximately 12-15 subjects, including replacement of drop-outs

Study Population

Inclusion Criteria

1. Diagnosis of primary open angle glaucoma (POAG) confirmed by ophthalmological examination
2. Age 18-80 years
3. Subject has given written informed consent
4. Subject is scheduled for trabeculectomy with MMC (independent of study participation) due to treating physician's decision, as a result of
5. no longer tolerating available pharmacologic treatment of glaucoma or progressing despite pharmacologic treatment of glaucoma

Exclusion Criteria

1. History of any other form of glaucoma than POAG in either eye
2. History of relevant ocular trauma in either eye < 6 months prior to Screening
3. History of ocular infection or ocular inflammation in either eye < 3 months prior to Screening
4. History of chronic or recurrent severe inflammatory eye disease, any severe ocular pathology, or clinically relevant or progressive retinal diseases in either eye
5. Clinically relevant, severe central visual field loss, or documented significant progression of a visual field defect within 6 months prior to Screening in either eye unrelated to glaucoma
6. Planned operation on the contra-lateral eye within the next 3 months
7. Use of any ophthalmic medication or substance on a chronic basis which has not been taken at a stable dose for at least 14 days prior to Screening
8. The presence of any concurrent condition or clinically significant laboratory findings at Screening that may interfere with any aspect of safety, study conduct, or interpretation of results
9. Known active HIV, chronic Hepatitis B or C infection or active Tuberculosis
10. History or evidence of any other clinically significant disorder, condition or disease (with the exception of the study disease as outlined above) that, in the opinion of the investigator or Isarna Therapeutics physician, if consulted, would pose a risk to subject safety or interfere with the study evaluation, procedure or completion
11. Pregnant or nursing women, sexually active male and female patients of childbearing potential not willing to use an effective form of contraception during participation in the study and for least 3 months after the last dose of the study medication
12. Subject likely to not be available to complete all protocol-required study visits or procedures, including follow-up visits, and/or to comply with all required study procedures to the best of the subjects and Investigator's knowledge
13. Currently receiving treatment in another investigational study or less than 30 days since ending treatment on another investigational study. Thirty days is calculated from Day 1 of protocol-specified therapy

Treatment Plan

Dosage and Administration

Patients will be enrolled in sequential cohorts into the study. The following dose levels of ISTH0036 are planned for exploration: 6.75 µg, 22.5 µg, 67.5 µg, and 225 µg per injection, equivalent to an approximate drug concentration of 0.3, 1, 3, and 10 µM in the vitreous body. The injection volume will be 50 µl per injection, for all dose levels.

A cohort review committee (CRC) meeting will be held after completion of the DLT period of all patients per cohort, after occurrence of DLT (to confirm) if necessary and after occurrence of any clinically relevant event deemed to interfere with the safety of study patients as per Investigator or Sponsor view. The CRC is responsible for review of data and decision on dose escalation, and all other key events related to patient safety.

Pharmaceutical-technical details and instructions for use of ISTH0036 will be provided by the sponsor.

Concomitant Medication

All concomitant medication should be recorded in the Case Report Form. If required, supportive therapy should be administered as medically needed in accordance with standard practice.

Duration of Subject Participation

The duration of study participation:

Core study: maximum 18 weeks (up to 6-week screening period including washout according to trabeculectomy standard procedure, single dose followed by a 6-week DLT period and a further 6-week follow-up period.

Post-study follow-up: until 1 year after End of Study visit.

Termination of treatment with ISTH0036 and/or termination of any further follow-up examinations:

Treatment with the investigational product should be discontinued and/or the subject withdrawn from the study in the event of any of the following:

- Withdrawal of subject's consent
- Subject or investigator not compliant with the study protocol
- Appearance of dose limiting toxicity (DLT)
- Apparent clinical glaucoma progression
- Investigator's decision that a change of therapy is in the subject's best interest
- Occurrence of an adverse event which makes discontinuation desirable or necessary in the investigator's and/or the subject's opinion
- Progression of a medical condition which in the opinion of the investigator should preclude further participation of the subject in the study
- In any case of premature treatment discontinuation, the investigator should make every effort to perform all examinations scheduled for the safety follow-up
- Prior to discontinuation of a subject, all examinations scheduled for the End of Study Visit should also be performed to allow for the evaluation of the study endpoint

Measurement and Evaluations

Each subject will have a minimum of 7 regular scheduled visits: 1 screening visit, 1 visit during the treatment period, 2 follow-up visits and 3 post-study follow-up visits.

Study assessments:

All subjects will have a medical history and concomitant medication assessment at Screening, followed by a continuous assessment of adverse events and concomitant medications during the study. A panel of safety laboratory evaluations (clinical chemistry, hematology and coagulation) and vital signs shall also be conducted at Screening, then at regular intervals as listed in the study flow charts in Appendix 1.

Follow up visits after the last dose of ISTH0036 will be performed after 42 days (6 weeks/Follow Up Visit) and 84 days (12 weeks/End of Study Visit). Both will include a full physical examination, vital signs, ophthalmologic examinations and safety lab investigation, plus AE and concomitant medication evaluation.

Safety

- Type and Frequency of Adverse events

Efficacy

- IOP
- Number of interventions post trabeculectomy
- Bleb filtering and morphology evaluation
- Visual acuity
- Visual field
- Slit lamp biomicroscopy
- Optic disc status (Heidelberg Retinograph II and photograph of the optic disc)

Statistical Methods

Sample Size

The sample size is not based on any statistical methodology but is considered sufficient to adequately investigate the objectives of the study.

Statistical Analysis

There will be no inferential statistical analysis in this study. Results will be presented using descriptive statistics. All subjects who receive at least one dose of ISTH0036 will be included in the safety analysis.

2 INTRODUCTION

2.1 Background

In ophthalmology, several diseases have been associated with modulation of transforming growth factor beta (TGF-β) protein expression. In particular, a large body of scientific evidence has been provided for glaucoma (Prendes *et al.*, 2013), proliferative vitreoretinopathy (Connor *et al.*, 1989; Kon *et al.*, 1999), posterior capsule opacification (Wormstone, 2002), and corneal diseases such as pterygium (Kria *et al.*, 1996) and keratoconus (Engler *et al.*, 2011).

Furthermore, single TGF-β isoforms of the TGF-β family (TGF-β1, -β2 and -β3) seem to be the core pathophysiologic molecular 'driving force' for various key ophthalmic diseases with high unmet medical need. Specifically for TGF-β2, a critical role in the pathophysiology of glaucoma, the second leading cause for blindness in the Western world, has been demonstrated, making this isoform an obvious therapeutic target of high interest.

TGF-β2 is the predominant cytokine in the eye (Freedman and Iserovich, 2013; Saika, 2006) and is found in large amounts in the aqueous and vitreous humors, the neuronal retina and the retinal pigmented epithelium in the healthy eye (Granstein *et al.*, 1990; Jampel *et al.*, 1990; Pfeffer *et al.*, 1994). In trabecular meshwork and optic nerve head, TGF-β2 is also present, but only expressed in small amounts (Pasquale *et al.*, 1993; Pena *et al.*, 1999; Zode *et al.*, 2011) (Tovar-Vidales *et al.*, 2011). Various studies have shown the potential significance of TGF-β2 signaling by observing that active TGF-β2 protein is significantly increased intraocularly in primary open-angle glaucoma (POAG) patients (Inatani *et al.*, 2001; Min *et al.*, 2006; Ochiai and Ochiai, 2002; Ozcan *et al.*, 2004; Picht *et al.*, 2001; Schlotzer-Schrehardt *et al.*, 2001; Tripathi *et al.*, 1994; Trivedi *et al.*, 2011; Yamamoto *et al.*, 2005). This effect is also visible in the optic nerve head, where TGF-β is increased to levels 70- to 100-fold above those of normal individuals (Pena *et al.*, 1999). TGF-β2 protein present in the aqueous humor is released by the epithelial layers of ciliary body and lens (Allen *et al.*, 1998; Gordon-Thomson *et al.*, 1998; Helbig *et al.*, 1991; Wallentin *et al.*, 1998). Other tissues in the posterior part of the eye also produce TGF-β2, such as retinal cells, hyaluronic cells and the dorsal epithelial cells of the lens. TGF-β2-induced changes might contribute to deformation of the optic nerve axons by causing impairment of axonal transport and neurotrophic supply, leading to their permanent degeneration. The increase in intraocular pressure further adds mechanical stress and strain to optic nerve axons and accelerates degenerative changes (Quigley, 2011).

TGF-β also plays a distinct driving role in fibrotic diseases (Border and Noble, 1994) and epithelial-mesenchymal transition and is therefore most probably responsible for the increase of extracellular matrix (ECM) and cellular transformation which is reported for the trabecular meshwork in glaucoma patients (Rohen and Witmer, 1972; Tektas and Lütjen-Drecoll, 2009). Recent studies indicate that the action of TGF-β2 on trabecular meshwork ECM synthesis requires, as a downstream mediator, connective tissue growth factor (CTGF), which is a member of the CCN (CYR61/CCN1, CTGF/CCN2, NOV/CCN3) family of matricellular regulatory proteins (Ihn, 2002; Phanish *et al.*, 2009). CTGF is strongly and constitutively expressed in the trabecular meshwork in situ (Tomarev *et al.*, 2003) and is markedly induced in cultured trabecular meshwork cells upon treatment with TGF-β2 (Bollinger *et al.*, 2011; Fuchshofer *et al.*, 2007). The action of TGF-β2 on the trabecular meshwork in POAG appears to be mainly mediated through CTGF (Bollinger *et al.*, 2011; Fuchshofer *et al.*, 2007; Ihn, 2002; Phanish *et al.*, 2009; Tomarev *et al.*, 2003), whereas the activities of TGF-β1 and -β2 are modulated by the blocking effects of bone morphogenetic protein-4 (BMP-4) and BMP-7, Smad7, and gremlin that inhibits BMP signaling as well as several species of microRNAs (Fuchshofer, 2011; Fuchshofer and Tamm, 2012).

Plasminogen activator inhibitor (PAI)-1 is also elevated in POAG patients and is an inhibitor of a class of proteolytic enzymes, called matrix metalloproteinases (MMPs) and is downstream of TGF-β2 (Fuchshofer *et al.*, 2003). In addition, LTBP2, the gene encoding latent TGF-β binding protein 2, was

identified in 2009 as a gene causing primary congenital glaucoma (Ali *et al.*, 2009), further supporting the critical role of the TGF-β pathway in glaucoma.

Furthermore, it has been previously demonstrated that intraocular administration of TGF-β isoform selective antisense oligonucleotides significantly prolong bleb survival in experimental preclinical model of glaucoma (Cordeiro *et al.*, 2003).

Glaucoma, the second leading cause for blindness in the adult in the Western world, is a progressive optic neuropathy characterized by gradually increasing loss of retinal ganglion cells, which manifests clinically with loss of optic disc neuroretinal rim tissue, defects in the retinal nerve fiber layer, and deficits on functional visual field testing (Danesh-Meyer *et al.*, 2006). Glaucoma is considered to be caused mainly by chronically increasing intraocular pressure. Despite a multitude of treatment options, including surgical procedures in refractory patients, blindness remains a major threat. Worldwide in the year 2020 the number of people with POAG is estimated at nearly 59 million, with 5.9 million experiencing bilateral blindness (Quigley and Broman, 2006). Primary open-angle glaucoma which accounts for the majority of glaucoma cases (~ 90%) mainly results from impaired drainage of aqueous humor out of the eye via the trabecular meshwork and/or uveoscleral pathways (Congdon *et al.*, 1992). The currently available treatment modalities are mainly focusing on reduction of intraocular pressure (Damiano *et al.*, 2007) by targeting the physiologic aqueous humor dynamics. The medical armamentarium includes prostaglandin analogues, beta blockers, alpha-agonists, carbonic anhydrase inhibitors, parasympathomimetics and hyperosmotics (Sambhara and Aref, 2014). Yet, many patients become refractory to the existing medications, experience increasing intraocular pressure despite all medication received, or experience adverse events related to certain medications that do not allow their use anymore.

When target intraocular pressure (Damiano *et al.*) can no longer be achieved by medical intervention, laser or incisional surgical interventions may be indicated. Among conventional external filtering operations for glaucoma, trabeculectomy is the most commonly performed surgical intervention and remains the standard of care for patients who have failed maximal tolerated medical therapy (Bettin and Di Matteo, 2013). With this surgical technique, an artificial canal is created between the anterior chamber and the subconjunctival space. The main threat to post-surgical failure is excessive wound healing of the conjunctiva and Tenon's capsule and scarring, processes in which TGF-β is known to play a major role (Georgoulas *et al.*, 2008). Subconjunctival fibrosis and the underlying mechanism of myofibroblast transformation are triggered by vascular endothelial growth factor (VEGF) which induces TGF-β1 expression (Park *et al.*, 2013). Anti-scarring agents have therefore found their way into clinical practice, even though most of their use is off-label. Even though these antimitotics, 5-fluorouracil (5-FU) and mitomycin C (MMC), have improved surgical success by improving bleb survival to some degree, they can potentially bring along severe vision-impairing side effects such as corneal toxicity, blebitis, endophthalmitis and hypotony (Van Bergen *et al.*, 2014), (Lockwood *et al.*, 2013).

Consequently, it appears desirable to (1) create novel treatment opportunities for patients that do no longer benefit from available standard medication and (2) improve the treatment situation of those patients that undergo trabeculectomy, by providing novel treatments that preserve the benefit of the surgical intervention. In light of the known pathophysiology, antagonists of TGF-β could provide such treatment alternatives.

2.2 Investigational Product

The antisense oligonucleotide ISTH0036 is 100% homologous to TGF-β2 mRNA of humans, rabbit, mouse, dog, rat, cynomolgus and rhesus monkey, and pig.

Upon Watson-Crick base pairing of the antisense oligonucleotide with the target mRNA, the strands are cleaved by RNase H. RNase H is an ubiquitous enzyme that hydrolyzes the RNA strand of an RNA/DNA duplex (Stein and Hausen, 1969). Oligonucleotide-assisted RNase H-dependent reduction of targeted

RNA expression can be quite efficient, reaching 80–95% downregulation of protein and mRNA expression.

The drug product contains 6.75 mg anhydrous ISTH0036 sodium salt/vial expressed as total oligonucleotide content. Immediately prior to administration, the lyophilized powder is reconstituted aseptically in isotonic (0.9%) saline solution for intravitreal injection to achieve the concentrations according to the clinical trial protocol.

2.3 Summary of Non-clinical Studies

Safety evaluation of ISTH0036 was conducted in pigmented Dutch-Belted and non-pigmented New Zealand White rabbits, Beagle dogs and Cynomolgus monkeys with single and repeated intravitreal (IVT) or intravenous administration.

The toxicity of ISTH0036 was tested in the rabbit following three intravitreal (IVT) administrations at 2-week intervals followed by a 3-month drug-free observation period (ISRN-PRD-1443). Repeated IVT injection of ISTH0036 at doses of 6.75, 20, 67.5 and 202 µg/eye/administration (resulting in calculated drug concentrations of about 1, 3, 10 and 30 µM in the vitreous humor) was not associated with any treatment-related premature sacrifice during the study. Observed findings consisted of slight to marked diffuse opacities of the posterior capsule of the lens, white areas in the vitreous body and marked horizontal white line in the vitreous, as detected in control and ISTH0036 treated animals, with a comparable incidence. These observations are described as possible consequences of the IVT administrations and compound-related effects were excluded. Focal to diffuse opacities of the anterior capsule and the nucleus of the lens, observed unilaterally or in both eyes, were clearly related to the treatment with ISTH0036 at the high dose level (202 µg/eye to reach a calculated drug concentration of 30 µM in the vitreous humor) since these uncommon findings in the rabbit affected the 202 µg/eye animals only (from day 40 onwards). ISTH0036-related histopathological effects were observed in the lens of the left eye in 3/10 animals treated with 202 µg ISTH0036 per eye. The lesions were mainly located at the equatorial region of the lens and were characterized by enlargement, disarrangement, and vacuolation of the cytoplasm of epithelial cells. These changes were accompanied by the presence of an epithelial layer beneath the thin posterior capsule of the lens, composed of flattened epithelial cells with elongated nuclei. At the end of the treatment-free observation period, a more severe disorganization of the lens fibers was observed in 11/12 animals previously given 202 µg ISTH0036 per eye, which also showed an opaque appearance of the lens at the ophthalmological evaluation during the in-life phase. In one female given 202-µg ISTH0036 per eye and showing severe test item-related disorganization of the lens fibers, there was a diffuse detachment and atrophy of the retina in the eye. Any relationship to treatment is considered unlikely and it could not be excluded that the retinal detachment and atrophy were secondary to the administration procedure.

The results at the various dose levels in the GLP rabbit toxicity study (3 x IVT injection at 2-week intervals) are summarized below.

Table 1: Summary of preclinical toxicity assessments of the 4-week toxicology study in Dutch-Belted rabbit

	Dose per eye / concentration	6.75 µg<br>1 µM	20 µg<br>3 µM	67.5 µg<br>10 µM	202 µg<br>30 µM
Rabbit<br>3x IVT<br>(2 week intervals)	Ophthalmic Observations	NDO<br>No ophthalmic exam after Day 30	NDO	NDO<br>No ophthalmic exam after Day 30	Lens opacification as of Day 40
	Histopathology	NDO<br>No exam after Day 30	NDO at Day 30 and week 16	NDO at Day 30<br>No exam after Day 30	Lesions at the equatorial region of the lens at Day 30 and at week 16
	ERG in week 6/7 and week 16	No ERG exam	NDO in week 6/7 and week 16	No ERG exam	Changes observed in week 6/7. No exam in week 16
NDO: no drug related adverse observations; ERG = electroretinogram					

Alterations of the electroretinogram (ERG), including increase in the implicit time and/or decrease in amplitude were observed in animals treated at 202 µg/eye when analysed 2-3 weeks after the last dose (week 6/7). At this dose level, in photopic conditions, changes consisted of decrease in b/a wave ratio up to 40% in males, when compared with pre-test data. In scotopic conditions, the same phenomena was observed in both sexes with a decrease of 46% in males and 33% in females. The absence of discernible response in some animals after 20 minutes of dark adaptation is a major abnormality showing that rods did not react to a light stimulus. This abnormality was observed at the highest dose level only, in 4/6 males and 4/6 females. No ERG changes were observed at the 20 µg/eye (3 µM) dose level (no ERGs were performed in the 6.75 µg/eye and 67.5 µg/eye group) in week 6/7, week 10/11 and week 14/15.

A 4-week ocular tolerance study of ISTH0036 in New Zealand White rabbits revealed that a single IVT injection with ISTH0036 at 6.5, 65 or 650 µg/eye/dose (to reach a calculated drug concentration of 1, 10 or 100 µM in the vitreous humor, respectively) was generally well-tolerated with only a dose-associated posterior segment inflammatory response occurring at the 650 µg/eye/dose. In animals receiving 650 µg/eye/dose, i.e. at a 100 µM dose retinal degeneration was noted on Day 15 in 2/3 animals and moderate posterior segment inflammation was observed in all three animals on Day 29. Animal models for safety studies show the expected pharmacological effects according to the presumed mechanism of action. Therefore, the animal models are regarded as relevant for prediction of adverse effects in human beings.

Limited preliminary tolerance study in Beagle dog indicated that single IVT administration of ISTH0036 at 300 µg/eye (corresponding to 30 µM initial concentration in the vitreous humor) did not induce any drug related changes when analyzed at Day 35 (ISRN-PRD-1457a).

To support repeated dosing in human, a 9-month toxicology study in Dutch-Belted rabbits followed by a 12-week treatment free observation was performed. The toxicity of ISTH0036 was assessed after 11 IVT injections administered once every 4 weeks in male and female Dutch-Belted rabbits (ISRN-PRD-1501).

Seventy-two rabbits were assigned to four dose groups, each consisting of 9 rabbits of each sex. ISTH0036 was administered by intravitreal injections at dose levels of 6.75, 20 or 67.5 µg/eye (corresponding to calculated drug concentrations of 1, 3 and 10 µM in the vitreous humor).

Dutch-belted rabbits did not reveal signs of systemic toxicity but almost all animals exhibited clinical manifestations of ISTH0036 induced cataractogenic process, as evidenced by opacities of the lens with a dose and time-related increased severity and incidence. Histologically, lens disorganization,

characterized by enlargement and fragmentation of the fibers at the equatorial region and beneath the posterior and anterior capsule were observed in the 20 and 67.5 µg/eye dose group. ERG examination revealed ISTH0036-related changes associated to retinal dysfunction in photopic and scotopic conditions at the highest dose level only. The ophthalmic and histopathological adverse findings did not regress at the end of the treatment-free period. No ERG changes were observed at 6.75 and 20 µg/eye dose levels.

Furthermore, an exploratory 28-week safety and toxicokinetic study of ISTH0036 in Cynomolgus monkeys followed by a 12-week treatment free observation period was performed (ISRN-PRD-1502).

Ten monkey were assigned to five dose groups, each consisting of 1 monkey of each sex. ISTH0036 was administered by intravitreal injections every 4 weeks (5 injections). Group 1 served as a control (vehicle-treated group), group 2-5 received 30, 100, 300, and 1000 µg/eye/administration (resulting in calculated drug concentrations of 3, 10, 30 and 100 µM in the vitreous humor) respectively. No signs of systemic toxicity were observed. Animals treated with 1000 µg/eye showed severe cortical cataract and a retinal degeneration, which resulted in severely reduced amplitudes of the ERG. Corneal edema was observed in this dose group. Histological findings in lens included formation of Morgagnian globules within cortical lens fibers, fluid filled spaces between cortical lens fibers, vacuolation of cortical lens fibers and detachment of the lens epithelium. Additional microscopic findings were observed in the retina and iris. Animals treated with 300 µg/eye showed marked to severe cortical cataract, with no evidence for retinal degeneration or vacuolation in the iris. The ERG was not affected. In animals treated with 100 µg/eye, slight to moderate cataract was observed. In animals treated with 30 µg/eye, mild cataract was noted, microscopically characterized by a minimal swelling of lens fibers.

Several *in vivo* pharmacology studies performed with ISTH0036 upon intra-ocular injection(s) in mice demonstrated significant sequence-specific increase in bleb size and survival post-operative filtration surgery (glaucoma filtration surgery model) and marked decrease in the level of neoangiogenesis in choroid neovascularization model post laser-induced burns. These preliminary results provide strong rationale for biologically relevant response to intra-ocular administration of ISTH0036.

For all further preclinical studies, please refer to the Investigator's Brochure ISTH0036.

2.4 Summary of Clinical Studies

This is the first clinical study in humans.

2.5 Rationale for Dose Selection

Based upon a 4-week preclinical toxicology study in Dutch-Belted rabbits, the No Adverse Effect Level (NOAEL) was set at 3 µM final concentration in the vitreous humor. Consequently, a starting dose achieving the 0.3 µM level in the human vitreous humor has been selected, representing 1/10 of the NOAEL. For further details regarding dose selection refer to the Investigator's Brochure.

2.6 Known and Potential Benefits and Risks

2.6.1 Benefits

Benefits of treating glaucoma in the trabeculectomy setting could be multifold: (1) TGF-β2 is known to play a role in fibrotic reactions in the eye, consequently, by effective inhibition closure of the bleb may be prevented. (2) TGF-β2 has been described to have a direct pathophysiologic effect on the optic nerve, and inhibiting this activity could preserve the optic nerve in glaucoma patients. (3) TGF-β2 has been demonstrated to be a key-driver in trabecular meshwork pathology, which promotes IOP-rise and glaucoma. Blocking these effects could prevent bleb closure, optic nerve damage and rise of the intraocular pressure post trabeculectomy.

2.6.2 Risks

Known clinical risks apart from those associated with an intravitreal injection in general are not available due to lack of prior clinical experiences with ISTH0036.

Regarding potential risks of single administration of ISTH0036 the preclinical data analysis indicates that inhibition of TGF-β2 with ISTH0036 may result in lens opacification at dose levels above those intended to be tested in humans. This observation was made on Day 40 in 3/12 rabbit eyes after three intravitreal injections at 2-week intervals with 30 µM ISTH0036. At that high dose also changes in electroretinograms were observed. No other clinically relevant toxicities were observed at any doses.

Results of a 9-month toxicology study have revealed effects after 5 IVT injections administered once every 4 weeks in male and female Dutch-Belted rabbits. In this study with repeated administration of ISTH0036, rabbits started to show lens opacifications at doses of 1 µM. As lens opacifications were also observed in the vehicle group and as the rabbit eye is known to possess higher sensitivity as compared to other species, these findings must be interpreted with caution and may (at least in part) be related to the injection procedure itself. An exploratory 28-week toxicokinetic study in Cynomolgus monkey with 5 IVT injections administered once every 4 weeks, monkeys started to show mild cataract at doses of 3 µM.

Additional preclinical studies will further investigate these findings to explore the basis for the planned repeated dosing in a subsequent trial (long term exposure).

For further details reference to the Investigator’s Brochure is made. Clinical safety monitoring should show special attention to any such findings.

Potential risks associated with the procedure (intravitreal injection), such as bleeding, endophthalmitis, bacterial infections and similar findings are recognized risks for intravitreal injection and should be carefully monitored.

2.7 Study Rationale

The cytokine human TGF-β is known to play a key role in various ophthalmic diseases. In glaucoma, increased levels of TGF-β2 present in the eye have been linked to trabecular meshwork transformation, increased intraocular pressure and direct optic nerve damage.

In advanced stage glaucoma trabeculectomy is the standard surgical intervention to reduce intraocular pressure in subjects not responding sufficiently anymore to pressure-lowering medications. Yet, scarring of the surgically opened canal (“bleb-closure”) often abolishes the effect of trabeculectomy (despite the intraoperative use of Mitomycin C to prevent this) and the surgical intervention itself does not block core glaucoma pathophysiologic processes. Importantly, TGF-β2 has been described to play a distinct role in the fibrotic process of bleb closure.

Consequently, blocking the effect of TGF-β2 by a selective antisense oligonucleotide (ASO) in the context of trabeculectomy appears to be an attractive potential therapeutic concept to (1) prevent bleb closure, (2) block any key pathophysiology of TGF-β2 in glaucoma progression (trabecular meshwork transformation with IOP-rise and optic nerve damage).

ISTH0036 is a ‘next generation’ antisense oligodeoxynucleotide based on locked-nucleic acid technology (LNA), selectively targeting TGF-β2, with favorable tissue penetration and tissue half-life. By forming complexes with TGF-β2 isoform mRNA, ISTH0036 blocks TGF-β2 protein production and results in degradation of the respective TGF-β2 isoform mRNA. Preclinical data have shown that TGF-β2 ASO has potent glaucoma progression preventing activity in various in vitro and in vivo preclinical models. Preclinical toxicology studies have shown that treatment with ISTH0036 is safe and overall well tolerated.

In summary, exploration of the ISTH0036 in the trabeculectomy setting appears justified and of high medical interest. This is the First-in-Human trial of ISTH0036.

3 STUDY OBJECTIVES

3.1 Primary Objective(s)

To determine the safety and tolerability of ISTH0036

3.2 Secondary Objective(s)

To determine preliminary clinical efficacy of ISTH0036

4 INVESTIGATIONAL PLAN

4.1 Study Endpoints

4.1.1 Primary Endpoint(s)

- Type and frequency of adverse events

4.1.2 Secondary Endpoint(s)

All parameters will be compared to baseline (Day -1) or screening, as applicable. On ISTH0036 treatment days, all examinations will be done pre-dose.

Table 2: Secondary endpoints and corresponding methods of assessment

Secondary endpoint	Methods of assessment	Time point
IOP	Goldmann tonometer	FU, EOS<br>PSFU1*, PSFU2*
Number of interventions post trabeculectomy	Documentation of surgical and non-surgical interventions	Whole post-operative study period up to EOS
Bleb filtering and morphology	Wuerzburg Bleb Classification	Day 3, FU, EOS
Visual acuity	Early Treatment Diabetic Retinopathy Study (ETDRS) chart; log MAR scoring system	Day 3, FU, EOS
Visual field	Humphrey Standard 24-2 or 30-2 or Octopus Standard 24-2 or 30-2 program	FU, EOS
Slit lamp biomicroscopy	Slit lamp biomicroscopy	FU, EOS
Optic disc status	Heidelberg Retina Tomograph II (HRTII)<br>Photograph of the optic disc	FU, EOS<br>FU, EOS

FU = Follow-up; EOS = End of study; PSFU = Post-study follow-up; * exploratory long-term evaluation

4.2 Study Design

This is a phase I, open-label, dose-escalation study to investigate the safety, tolerability and clinical activity of ISTH0036, a 'next generation' TGF-β2-selective ASO. This compound is intended for the treatment of glaucoma patients in advanced disease stage that have exhausted available medication options, and are undergoing glaucoma filtration surgery (trabeculectomy). The compound is expected to block core pathophysiologic mechanisms that drive glaucoma progression, e.g. trabecular meshwork alteration, and provide potential optic nerve protection.

Single dose, dose escalation study to explore the safety and tolerability of single doses of ISTH0036. Patients will be enrolled in cohorts of three patients to evaluate each dose level administered. Up to 4 different dose levels of ISTH0036 will be evaluated. The study medication will be administered as an intravitreal injection on Day 1 (during surgery, at the end of the trabeculectomy surgical procedure, after topical MMC administration). There will be a minimum interval of 1 week between the dosing of the first and all subsequent patients in each cohort. Dose escalation will occur when data from 3 subjects who have completed the dose limiting toxicity (DLT) monitoring period of 42 days is available and has been reviewed by the Cohort Review Committee (CRC) and no DLT has been observed.

All dose escalation steps will be recommended and approved by the CRC who will convene to review all available AEs, laboratory and other relevant subject data on a regular basis prior to dose escalation decision making and at other timepoints if considered necessary by the CRC. See Appendix 2 for the Cohort Review Committee process.

The study medication will be administered on Day 1 (during surgery, at the end of the trabeculectomy surgical procedure, after MMC administration). The pre-operative, surgery and post-operative care of the subject will follow the standard procedure described in the standard operating procedure (SOP) in Appendix 4. The SOP for the intravitreal injection is described in Appendix 5.

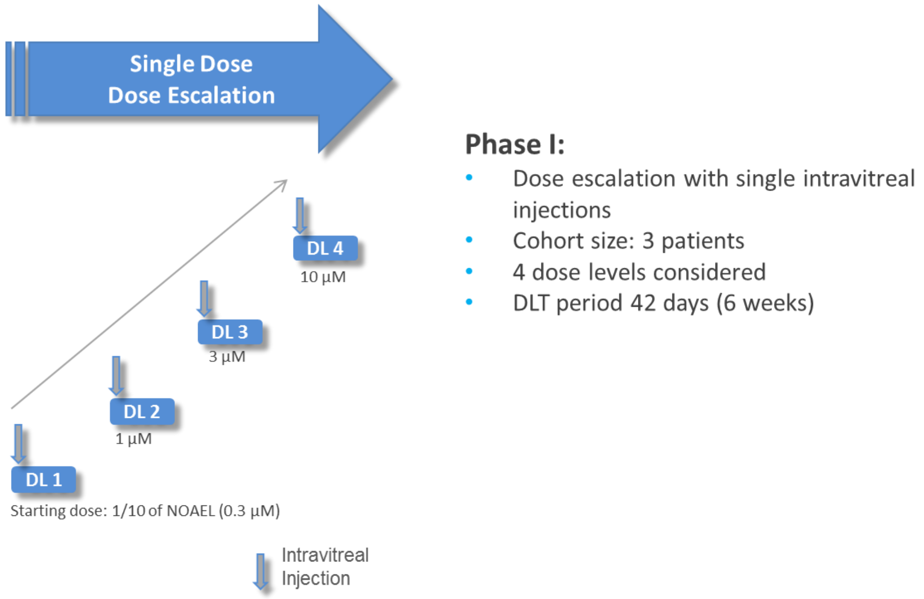

Safety will be reviewed on an ongoing basis (adverse events [AEs], concomitant medication, safety lab and ophthalmological examinations)and will be discussed during the cohort review process. Clinically significant events thought to be potentially related to ISTH0036 administration, or any significant AE profile changes seen , will be taken into account when considering future dose escalation steps.

DLTs and AEs will be graded for severity based on the National Cancer Institute Common Terminology Criteria for Adverse Events version 4.03 (NCI CTCAE v4.03) (Appendix 8). Examples of toxicity events, which qualify to be dose-limiting, are listed in Section 7.2 and must be considered at least possibly related to treatment with ISTH0036.

5 STUDY POPULATION

5.1 Inclusion Criteria

A subject will be eligible for study participation only if all of the following criteria apply:

1. Diagnosis of primary open angle glaucoma (POAG) confirmed by ophthalmological examination
2. Age 18-80 years
3. Subject has given written informed consent
4. Subject is scheduled for trabeculectomy with MMC (independent of study participation) due to treating physician's decision, as a result of
5. no longer tolerating available pharmacologic treatment of glaucoma or progressing despite pharmacologic treatment of glaucoma

5.2 Exclusion Criteria

A subject will not be eligible to participate in this study if any of the following criteria apply:

1. History of any other form of glaucoma than POAG in either eye
2. History of relevant ocular trauma in either eye < 6 months prior to Screening
3. History of ocular infection or ocular inflammation in either eye < 3 months prior to Screening
4. History of chronic or recurrent severe inflammatory eye disease, any severe ocular pathology, or clinically relevant or progressive retinal diseases in either eye
5. Clinically relevant, severe central visual field loss, or documented significant progression of a visual field defect within 6 months prior to Screening in either eye unrelated to glaucoma
6. Planned operation on the contra-lateral eye within the next 3 months
7. Use of any ophthalmic medication or substance on a chronic basis which has not been taken at a stable dose for at least 14 days prior to Screening
8. The presence of any concurrent condition or clinically significant laboratory findings at Screening that may interfere with any aspect of safety, study conduct, or interpretation of results
9. Known active HIV, chronic Hepatitis B or C infection or active Tuberculosis
10. History or evidence of any other clinically significant disorder, condition or disease (with the exception of the study disease as outlined above) that, in the opinion of the investigator or Isarna Therapeutics physician, if consulted, would pose a risk to subject safety or interfere with the study evaluation, procedure or completion
11. Pregnant or nursing women, sexually active male and female patients of childbearing potential not willing to use an effective form of contraception during participation in the study and for least 3 months after the last dose of the study medication
12. Subject likely to not be available to complete all protocol-required study visits or procedures, including follow-up visits, and/or to comply with all required study procedures to the best of the subjects and Investigator's knowledge
13. Currently receiving treatment in another investigational study or less than 30 days since ending treatment on another investigational study. Thirty days is calculated from Day 1 of protocol-specified therapy

6 INVESTIGATIONAL MEDICINAL PRODUCT

6.1 Description of the Investigational Medicinal Product

6.1.1 Formulation, Packaging and Labeling

The Investigational Medical Product will be provided as 6.75 mg sterile lyophilized ISTH0036 sodium salt for reconstitution filled in 6R glass vials. Labels will be designed in compliance with the respective

national legislation applicable for the country participating in the study. The label may contain the following information:

Label for immediate container (vial):

- Name and address of the sponsor and Clinical Research Organization (CRO)
- Pharmaceutical dosage form, route of administration, quantity of dosage units, name/identifier of product
- Batch no.
- Trial reference code
- Subject identification
- Kit identification

The label for the outer packaging (box) will contain the following information:

- Name, address and phone number of the sponsor and CRO
- Pharmaceutical dosage form, route of administration, quantity of dosage units, name/identifier of product
- Batch no.
- Trial reference code
- Subject identification
- Kit identification
- Name of the investigator
- Direction for use
- For clinical trial use only
- Storage conditions
- Period of use (Expiry date)

Additional information will be included on the labels as required per the respective national regulatory authorities.

For Germany (box label):

- EudraCT no.
- Statement about handling of used drug

Information on the label will appear in the official language(s) of the country in which the investigational medicinal product (IMP) is used.

If shelf-life of the product is extended during the trial, relabeling of IMP at the Trial Sites/Pharmacies may be performed according to local regulations, and must follow detailed instructions provided in the Study Reference Manual.

6.1.2 Storage and Stability

ISTH0036 drug product must be stored at 5 ± 3°C in their original secondary packaging within a secure environment, protected from light and separated from other medication or investigational product.

Reconstituted vials of ISTH0036 should not be stored for longer than 4 hours at ambient temperatures and 24 hours between +2°C and +8°C before use.

For further details, please refer to the *Investigator's Brochure*.

6.1.3 Preparation

Detailed instructions regarding the reconstitution and preparation for administration will be provided to the respective compounding unit before the IMP will be administered for the first time (refer to *Study Reference Manual, as applicable*).

For definition of dose levels, estimated final concentrations immediately after injection were calculated assuming a volume of 4.5 mL of vitreous humor in the eye.

The drug product will be reconstituted as listed below:

1. **Cohort 1: 0.3 µM ISTH0036 (6.75 µg total dose)**
   6.75 mg drug product reconstituted in 5 ml 0.9% normal saline solution for injection. After mixing, 4.5 ml of solution will be withdrawn and discarded. A further 4.5 ml of 0.9% normal saline solution for injection will be added to achieve a final concentration of approximately 0.3 µM in the vitreous body, assuming a human vitreous humor volume of 4.5 ml.
2. **Cohort 2: 1 µM ISTH0036 (22.5 µg total dose)**
   6.75 mg drug product reconstituted in 3 ml 0.9% normal saline solution for injection. After mixing, 2.5 ml of solution will be withdrawn and discarded. A further 2 ml of 0.9% normal saline solution for injection will be added to achieve a final concentration of approximately 1 µM in the vitreous body.
3. **Cohort 3: 3 µM ISTH0036 (67.5 µg total dose)**
   6.75 mg drug product reconstituted in 2.5 ml 0.9% normal saline solution for injection. After mixing, 1.5 ml of solution will be withdrawn and discarded. A further 1 ml of 0.9% normal saline solution for injection will be added to achieve a final concentration of approximately 3 µM in the vitreous body.
4. **Cohort 4: 10 µM ISTH0036 (225 µg total dose)**
   6.75 mg drug product reconstituted in 1.5 ml 0.9% normal saline solution for injection equivalent to a final concentration of approximately 10 µM in the vitreous body.

Table 3: Planned dose levels and corresponding reconstitution

Final concentration in the vitreous humor of the eye	Total dose in patients (50 µl)	Concentration in formulation	Amount saline for reconstitution
0.3 µM ISTH0036	6.75 µg	0.135 mg/ml	Add 5 ml, mix well, withdraw 4.5 ml from vial and discard.<br>Add 4.5 ml to achieve final concentration.
1 µM ISTH0036	22.5 µg	0.45 mg/ml	Add 3 ml, mix well, withdraw 2.5 ml from vial and discard.<br>Add 2 ml to achieve final concentration.
3 µM ISTH0036	67.5 µg	1.35 mg/ml	Add 2.5 ml, mix well, withdraw 1.5 ml from vial and discard.<br>Add 1 ml to achieve final concentration.
10 µM ISTH0036	225 µg	4.5 mg/ml	Add 1.5 ml, mix well.

For further details, please refer to the *Investigator's Brochure*.

6.1.4 Drug Accountability

Each study site will be supplied with a sufficient amount of IMP to treat the first subjects enrolled. Additional shipments of study medication will be performed according to the site's recruitment rate.

At each site, the pharmacist, or the person designated as the site‘s compounding person, is responsible for keeping accurate study drug accountability records, throughout the study, regarding the receipt of study medication, the dispensing of study medication to the study personnel and the return of all used and unused study medication. The drug accountability form will be periodically reviewed by the study monitor.

6.1.5 Destruction of IMP

The trial site shall return all unused IMP to the drug supplier for destruction or destroy it on site upon written authorization by the sponsor. Pursuant to sponsor's instruction. Used vials may be discarded at site in compliance with local procedures after drug accountability has been performed by the monitor. If tear-off labels are used for documentation, used vials must be kept until drug accountability has been checked by the monitor. In any case destruction of IMP and its documentation will be performed in accordance with Good Manufacturing Practice.

6.2 Description of the Placebo and/or Comparator

Not applicable

7 TREATMENT PROCEDURES

7.1 Treatment Schedule

Subjects will be enrolled in cohorts of three patients to evaluate each dose level administered. Up to 4 different dose levels of ISTH0036 will be evaluated. The study medication will be administered as an intravitreal injection on Day 1 (during surgery, at the end of the trabeculectomy surgical procedure, after topical MMC administration). There must be a minimum interval of one week between the dosing of the first and all subsequent subjects in each cohort. Dose escalation will occur when data from 3 subjects who have completed the DLT period of 42 days is available and all available safety data have been reviewed by the CRC and no DLT has been observed.

All subjects will receive standard post-operative treatment following the trabeculectomy.

7.1.1 Treatment Assignment

This is an open label study, eligible subjects will be assigned to the treatment in a sequential order.

Assignment of subject numbers:

Each site will be assigned a 2-digit center number e.g. 01, 02, 03 etc. Each subject screened and enrolled will be allocated a 4-digit subject number comprising the 2-digit center number and a 2-digit number representing the sequential order in which they are enrolled, e.g. 01-01, 01-02 etc.

7.1.2 Administration

50 µl of the reconstituted ISTH0036 will be injected into the vitreous humor of the target eye. See Appendix 5 for detailed instructions on the injection procedure for the intravitreal injection.

Standard operating procedure (SOP) for the trabeculectomy is provided in Appendix 4.

7.2 Definition of Dose Limiting Toxicity

Dose limiting toxicity is defined based upon the NCI CTCAE v4.03 (Appendix 8).

As DLT do qualify all toxicities that are declared to be at least possibly related to the study drug and

- are ≥ Grade 3 or
- are eye disorder toxicities ≥ Grade 2 or
- are any of the following toxicities ≥ Grade 1
    - Cataract
    - Retinal Detachment
    - Retinopathy.

Adverse Events that are seen as related to the pre-existing primary open angle glaucoma or the trabeculectomy with MMC procedure or other medical conditions and treatment interventions are not considered as DLT.

The DLT period is defined as the 42-day period following the first intravitreal injection.

Any DLT must be reported to the Sponsor using the DLT reporting form within 24 h after identification.

Any suspected DLT will be carefully evaluated for clinical relevance in communication with the medical representative of the Sponsor.

Observation of any DLT as confirmed by the Cohort Review Committee will lead to discontinuation of any further administrations at this dose level/in this cohort.

7.3 Subjects Compliance

Since the IMP is administered to the patients either by the investigator or his/her authorized personnel, no specific measures for patients' compliance control have to be taken. The administration of the IMP will be documented in the CRF and the documentation will be regularly reviewed by the study monitor.

7.4 Dose Interruption/Dose Modification for Adverse Events

There will be ongoing evaluation of AEs during treatment which will be discussed during the cohort review process. Clinically significant events thought to be potentially related to ISTH0036 administration, or trends in AEs seen in subsequent dosing, will be taken into account when considering future dose escalation steps. The CRC can decide to interrupt or terminate dosing of subjects at any time if seen as desirable by current safety data available. See attached Cohort review Committee process in Appendix 2.

7.5 Subject Discontinuation Criteria

Treatment with the investigational product should be discontinued and/or the subject withdrawn from the study in the event of any of the following:

Withdrawal of subject's consent

Subject or investigator not compliant with the study protocol

Appearance of DLT

Apparent clinical glaucoma progression

Investigator's decision that a change of therapy is in the subject's best interest

Occurrence of an adverse event which makes discontinuation desirable or necessary in the investigator's and/or the subject's opinion

Progression of a medical condition which in the opinion of the investigator should preclude further participation of the subject in the study

All reasons for treatment discontinuation should be clearly and concisely documented in the Case Report Forms. If a subject has not continued to present himself for study visits, the investigator should determine the reason and circumstances as completely and accurately as possible.

In any case of premature treatment discontinuation, the investigator should make every effort to perform all examinations scheduled for the safety follow-up. These data should be recorded, as they comprise an essential evaluation that should be performed prior to discharging any subject from the study.

Prior to discontinuation of a subject, all examinations scheduled for the End of Study Visit should also be performed to allow for the evaluation of the study endpoints.

7.6 Overdose

Overdose means that the subject was administered a higher dose of study medication than the dose(s) prescribed in this protocol.

In case of overdose, the subject should be monitored carefully and symptomatic treatment should be given as appropriate. If the overdose results in an AE, the subject should be followed up carefully until all signs of toxicity are resolved.

Overdose, with or without AE, should be handled as a serious adverse event (SAE) (see Section 11.4.2).

7.7 Concomitant Medication

The use of any other investigational study medication during the clinical trial is prohibited.

All concomitant medication, except general anesthesia medication used according hospital standards, should be recorded in the Case Report Form (CRF). Perioperative general anesthesia-related medication other than site’s standard regimen (i.e. different medication, exceptionally different dosage due to special medical conditions) should be recorded in the CRF. If required, supportive therapy should be administered as medically needed in accordance with standard practice.

8 STUDY PROCEDURES

An overview of all assessments at each visit can be found in Appendix 1.

8.1 Eligibility and Safety Assessments

Demographics

Date of birth, sex, height, weight and ethnic origin will be recorded at screening.

Medical History / Current Medical Conditions

General and disease specific medical history including a history of past and current medical conditions (including active HIV, chronic Hepatitis B or C, and active tuberculosis) and previous medication will be recorded at screening.

Physical Examination

Complete physical examinations of all body systems will be performed. All clinically relevant findings will be documented.

Vital Signs

Body temperature (oral or tympanic), heart rate and blood pressure (systolic/diastolic) will be measured at the times indicated in the flow charts.

Safety Laboratory Evaluations

Blood samples for safety laboratory evaluations will be taken at screening and as indicated in the flow charts. The following analyses will be performed:

Clinical Chemistry

Creatinine, lactate dehydrogenase (LDH), calcium, electrolytes (sodium and potassium), total bilirubin, alanine aminotransferase (ALAT), aspartate aminotransferase (ASAT), γ-glutamyl transferase (GGT), albumin, alkaline phosphatase, glucose.

Hematology

Red blood cell count (RBC), hemoglobin, hematocrit, white blood cell count (WBC), differential blood count, and platelet count.

Coagulation

International normalized ratio of prothrombin time (INR).

Urinalysis

Presence of glucose, protein and blood in urine will be assessed by dipstick.

Pregnancy Test

A serum pregnancy test (β-human chorionic gonadotropine [β-HCG]) will be performed in all women of child-bearing potential during the screening period. Absence of pregnancy will be confirmed (urine test may be used) on Day-1.

Concomitant Medication and Interventions

All concomitant medications and further medical interventions will be recorded.

Adverse Events

Adverse events occurring during the screening period (i.e. after obtaining the patient's written informed consent but prior to Day 1) will be recorded on the Medical History/Current Medical Conditions page in the CRF. AEs occurring during the treatment period (starting with Day 1) and until 12 weeks after the last IMP administration will be recorded on the Adverse Event Form in the CRF. During the screening period, AEs related to study procedures should also be reported. For reporting of AEs, please refer to Section 11.4.1.

Serious Adverse Events

Serious adverse events will be recorded on the Adverse Event Forms in the CRF during the entire study period, including the screening and the follow-up periods, with the box for "seriousness" ticked. For reporting of SAEs, please refer to Section 11.4.

Electroretinogram (ERG)

An ERG to measure the electrical response of the light-sensitive cells in the patient's eyes will be performed at screening and repeated at the follow up visit (FU).

8.2 Cohort Review Committee

The Cohort Review Committee will be the main platform for communication and discussion of all clinical information to ensure that new safety findings and all other relevant information are transmitted to all participating sites and that the integrity of the study design is not compromised.

The CRC is responsible for review of data and decision on dose escalation, or definition of doses where applicable.

The CRC will review dose limiting toxicities and all additionally emerging clinically relevant safety data for each patient, respective dose cohorts and overall. Therefore information on treatment tolerability will be provided to CRC after completion of the DLT period (42 days) of a patient and/or cohort, prior to meetings or immediately in case of important safety information.

The CRC meeting will be held after completion of the DLT period of all patients per cohort, after occurrence of DLT (to confirm) if necessary and after occurrence of any clinically relevant event that might interfere with the safety of study patients. For composition of the CRC and more details refer to Appendix 2.

8.3 Assessment of Efficacy

8.3.1 Visual acuity

The best corrected visual acuity will be measured for both eyes under normal room light using the Early Treatment Diabetic Retinopathy Study (ETDRS) chart and the logMAR scoring system.

8.3.2 Visual field

Visual field will be measured before ophthalmoscopy using the Humphrey Standard 24-2 or 30-2 or Octopus Standard 24-2 or 30-2 program.
The visual field test result will be reported as normal or abnormal. If abnormal it will be graded as mild, moderate or severe.

8.3.3 Slit-lamp biomicroscopy

The eyelids, conjunctiva, cornea, iris/anterior chamber and lens in both eyes will be examined by slit-lamp biomicroscopy. The examination will be performed before IOP measurements.

The findings will be reported as described in Appendix 3.

If there is a deterioration of two grades or more, the deterioration must be reported as an adverse event.

8.3.4 Bleb filtering evaluation

Bleb filtering will be evaluated by the investigator using slit lamp images. The bleb will be classified using the Wuerzburg Bleb Classification Score as described in Table 4.

Table 4: Wuerzburg Bleb Classification Score

Grade	3	2	1	0
Vascularization	Avascular	Similar to adjacent conjunctiva	Increased	massive
Corkscrew vessels	None	In one third	In two thirds	Entire bleb
Encapsulation	None	In one third	In two thirds	Entire bleb
Microcysts	Entire bleb	Lateral or medial of the flap	Over the scleral flap	none

(Picht and Grehn, 1998)

8.3.5 Intraocular Pressure Measurements

IOP will be measured in both eyes using the Goldmann tonometer.

The IOP measurements will be done in the sitting position. The fluorescein and anesthetic agent should be the same at each measurement.

Two consecutive measurements will be made for each eye and if the measurements differ by more than 2 mmHg a third measurement must be done. All measurements will be reported in the CRF and the median of the measurements will be considered the IOP for that eye.

Before use the tonometer must be checked for calibration accuracy according to the manufacturer's instructions and the tonometer must be calibrated monthly at the clinical trial site.

A SOP detailing the procedure is provided in Appendix 6.

Long-term IOP control for measurement of long-term effects during Post-Study Follow-up is ideally be done at the trial site using the Goldmann tonometer. Alternatively, data from IOP measurements performed by the subject's ophthalmologist according local standards can be collected.

8.3.6 Heidelberg Retina Tomograph (HRTII)

Confocal scanning laser ophthalmoscopy (CSLO) with Heidelberg Retina Tomograph II (HRTII) will be used to measure the stereometric parameters of the optic disc. The Moorefield's Regression Analysis and the Topographic Change Analysis will be used to detect progression. A SOP detailing the interpretation of the results is provided in Appendix 7. An instruction manual will be provided to each study site.

8.3.7 Optic disc photographs

Photographs will be taken to evaluate changes in the optic disc.

9 VISIT SCHEDULE

The visit schedule is summarized in Table 6 in Appendix 1. The calculation of all study days is based on Day 1, which is defined as the day of the dose of IMP.

9.1 Screening Period (Day -42 to Day -2)

Written informed consent will be obtained from each subject within a reasonable time prior to the performance of any study-related procedure.

All subjects, who have signed the study-specific Informed Consent Form (ICF), will be recorded on the Screening Log. Only those subjects who have met all eligibility criteria will be assigned a subject number (please refer to Section 7.1.1), and will be documented on the Screening and Enrolment Log. If a subject is screened but not enrolled in the study, the reason for screening failure should be recorded.

In order to ensure that the appropriate number of patients is enrolled to each cohort; on identifying a potential study patient, the Investigator is required to complete a patient registration request form confirming patient eligibility and requesting a place on a study cohort. Where appropriate, sites will then receive a completed patient registration request form confirming the enrolment of the patient and the cohort and dose that the patient is assigned to. Patients must not be enrolled until this confirmation is received.

Sexually active male and female patients of childbearing potential must agree to use an effective method of birth control (e.g. barrier methods with spermicides, oral or parenteral contraceptives and/or intrauterine devices) during the entire duration of the study and for 3 month after final administration of ISTH0036, or the patient must be surgically sterile (with documentation in the patient's medical records).

All screening evaluations must be performed between 2 and 42 days before the start of the treatment. All results should be available before a subject is declared eligible for study participation. Washout, if required, will be performed according to trabeculectomy standard procedure (refer to Appendix 4).

The following procedures and assessments should be performed during the screening period:

- Eligibility according to Inclusion/Exclusion Criteria (see Section 5)
- Demographics, height, weight
- Medical history/current medical conditions including medication
- Pregnancy test (in women of childbearing potential)
- Safety laboratory evaluation and urinalysis
- Physical examination
- Vital signs
- Visual acuity
- Visual field
- Slit-lamp biomicroscopy
- Intraocular pressure measurements
- Heidelberg Retina Tomograph II (HRTII) ophthalmoscopy
- Optic disc photographs
- Electroretinogram

9.2 Treatment Period

9.2.1 Visit 2 (hospitalization)

9.2.1.1 Day -1

The subject will be admitted to hospital approximately 24 hours before elected surgery for glaucoma.

The following assessments will be performed:

- Pregnancy test (in women of childbearing potential)
- Safety laboratory evaluation and urinalysis
- Vital signs
- Visual acuity
- Visual field
- Slit-lamp biomicroscopy
- Intraocular pressure measurements

The subject will be treated according to the standard procedures for pre-operative preparation for trabeculectomy. All concomitant medication and changes in health will be documented in the CRF.

9.2.1.2 Day 1

Vital signs will be measured before the trabeculectomy and 2 and 4 hours after the operation.

The trabeculectomy will be performed according to the standard procedure at the hospital. The investigator/surgeon will inject the first dose of ISTH0036 intraoperatively after completion of the trabeculectomy and after administration of MMC. Concomitant medication and adverse events will be documented.

9.2.1.3 Post trabeculectomy (Day 3)

The subject will be treated and remain in hospital according to the standard procedure at the hospital. The following assessments will be performed:

- Vital signs
- Visual acuity
- Bleb filtering evaluation
- Documentation of interventions post trabeculectomy
- Documentation of adverse events and concomitant medication

9.3 Follow-up Visit (FU)

Follow-up visits will be performed 6 weeks (FU) and 12 weeks (EOS) after the last injection of ISTH0036. This will be on Day 43+2 and Day 85±2. The EOS is described in Section 9.5. The following assessments will be performed at the FU visit on Day 43+2:

- Safety laboratory evaluation and urinalysis
- Physical examination
- Vital signs
- Visual acuity
- Visual field
- Slit-lamp biomicroscopy
- Bleb filtering evaluation
- Intraocular pressure measurements
- Heidelberg Retina Tomograph II (HRTII) ophthalmoscopy
- Optic disc photographs
- Electroretinogram
- Documentation of interventions post trabeculectomy
- Documentation of adverse events and concomitant medication

9.4 Unscheduled Visit(s)

Unscheduled visits should be performed whenever necessary, e.g. in case of adverse events or in case of symptoms of clinical disease progression.

Evaluations and/or assessments should be performed, as deemed appropriate by the investigator based on the nature of the event prompting an unscheduled visit.

The results of all examinations during an unscheduled visit should be documented in the subject's file and should be recorded in the CRF. If a subject is withdrawn from the study, all examinations scheduled for the End of Study Visit (Visit EOS) should be performed.

9.4.1 In Case of Adverse Events

The investigator will initiate appropriate treatment according to his/her medical judgment. The subject must be followed-up by additional examinations according to the medical judgment of the investigator, until the abnormal condition is resolved or the investigator deems further observations or examinations to be no longer medically indicated.

9.4.2 In Case of Signs and Symptoms of Disease Progression

If there is evidence of disease progression the investigator will perform any extra examinations and initiate treatment according to his/her medical judgment.

9.5 End of Study Visit (EOS)

The end of study visit should be performed 6 weeks after the last follow-up visit or if the subject is prematurely discontinued from the study to document all data relevant for evaluation of endpoints. The following assessments will be performed:

Safety laboratory evaluation and urinalysis

Physical examination

Vital signs

Visual acuity

Visual field

Slit-lamp biomicroscopy

Bleb filtering evaluation

Intraocular pressure measurements

Heidelberg Retina Tomograph II (HRTII) ophthalmoscopy

Optic disc photographs

Documentation of interventions post trabeculectomy

Documentation of adverse events and concomitant medication

9.6 Post-Study Follow-Up (PSFU)

To evaluate potential long-term effects of ISTH0036 on outcome of trabeculectomy and IOP values, post-study follow-up visits will be performed 6 months (PSFU 1) and 12 months (PSFU 2) after the last injection of ISTH0036 to collect IOP follow-up assessment data standardly performed either at trial site or at subjects's ophthalmologist and reported to trial site.

In order to capture any long term risks potentially related to the surgical procedure and/or the intravitreal administration of the study medication, patients will be contacted by phone 1 year after the End of Study Visit (PSFU 3) by their study physician or delegated staff and questioned about occurrence of any such events in the relevant period.

9.6.1 Post-Study Visit 6 months – PSFU 1

- Intraocular pressure measurements routinely performed at site/data collected from subject's ophthalmologist
- Documentation of any adverse events potentially related to the surgical procedures and/or the intravitreal administration of the study medication
- Documentation of concomitant anti-glaucoma medication

9.6.2 Post-Study Visit 12 months – PSFU 2

- Intraocular pressure measurements routinely performed at site/data collected from subject's ophthalmologist
- Documentation of any adverse events potentially related to the surgical procedures and/or the intravitreal administration of the study medication
- Documentation of concomitant anti-glaucoma medication

9.6.3 Post-Study Follow-Up (PSFU 3)

- Documentation of any adverse events potentially related to the surgical procedures and/or the intravitreal administration of the study medication

10 COLLECTION, STORAGE AND SHIPMENT OF LABORATORY SAMPLES

10.1 Safety Laboratory Assessments

All safety laboratory analyses (clinical chemistry, hematology, coagulation) will be performed at the local hospital laboratories.

11 DRUG SAFETY

The investigator is responsible for the detection and documentation of events meeting the definition of an AE or an SAE as provided in Section 11.1. This includes the evaluation of its seriousness, its severity, and the causal relationship to the investigational product and/or concomitant therapy (see Section 11.2).

At each visit, the investigator or co-investigator will assess whether any AE, including laboratory abnormalities, has occurred. The investigator should inquire for AEs with non-suggestive questions to the subject at each study visit.

The investigator should attempt to establish a diagnosis of the event based on signs, symptoms and/or other clinical information. The diagnosis should be recorded as the AE and/or SAE and not the individual symptoms.

If a laboratory abnormality or other abnormal assessment meet the definition of an AE or SAE, the Adverse Event Form or Serious Adverse Event Form should be completed as appropriate. A diagnosis, if known, or clinical signs and symptoms if diagnosis is unknown, rather than the laboratory abnormality should be completed on the CRF page. If no diagnosis is known and clinical signs and symptoms are not present, then the abnormal finding should be recorded.

The subject should be observed and monitored carefully until the AE resolves, the condition stabilizes or its cause is identified completely. The investigator is responsible to ensure that follow-up includes any supplemental investigations that may be indicated to elucidate the nature and/or the cause of the event.

11.1 Definitions

11.1.1 Adverse Events

An adverse event is defined as **any untoward medical occurrence in a subject or clinical trial subject administered a medicinal product and which does not necessarily have a causal relationship with this treatment**.

An adverse event can therefore be any unfavorable and unintended sign (including an abnormal laboratory finding), symptom, or disease temporally associated with the use of an investigational medicinal product, whether or not considered related to the investigational medicinal product.

The following events, detected or diagnosed during or after administration of the investigational medicinal product, are adverse events:

- A new sign, symptom, illness or syndrome,
- Aggravation (change in nature, severity or frequency) of a concomitant or pre-existing illness or permanent disorder,

  Marked hematological and other laboratory abnormalities, if judged clinically significant in the opinion of the investigator and any events that led to an intervention, including withdrawal of test drug/investigational product treatment, or significant additional concomitant therapy,
- An adverse effect of the investigational medicinal product or concomitant medication,
- Drug interactions,

- An adverse effect of an invasive procedure (e.g., complications resulting from invasive procedures) required by the protocol,
- An accident or injury.

The following events are not considered to be adverse events:

Medical or surgical procedures (e.g., surgery, endoscopy, tooth extraction, transfusion). However, the disorders, which led to the procedures, are AEs, if they did not exist before or if they worsened during or after administration of the investigational medicinal product,

Pre-existing diseases or conditions, which do not worsen during or after administration of the investigational medicinal product,

Hospitalization due to social reasons,

The disease under study or signs and symptoms associated with the disease, unless more severe than expected for the subject's condition.

11.1.2 Serious Adverse Event

A serious adverse event (experience) or reaction is any untoward medical occurrence or effect that at any dose:

Results in death, (1)

Is life-threatening, (2)

Requires inpatient hospitalization or prolongation of existing subject's hospitalization, (3)

Results in persistent or significant disability or incapacity (4),

Is a congenital anomaly or birth defect,

Is a medically important serious event (5).

In addition, all laboratory abnormalities of grade 4 severity that occur during or after administration of the investigational product should be reported as serious adverse event.

Comments:

1) Death is an outcome; the condition leading to death is the SAE.

2) The term "life-threatening" in the definition of a SAE refers to an event in which the subject was at risk of death at the time of the event; it does not refer to an event, which hypothetically might have caused death if it were more severe.

3.) This means that hospital inpatient admission or prolongation of hospital stay were required for the treatment of the adverse event, or that they occurred as a consequence of the event. Hospitalization signifies that the subject was in-subject for at least one overnight stay. Hospitalization for elective treatment of a pre-existing condition that did not worsen from baseline or hospitalization for social reasons are not considered SAEs.

4.) "Persistent or significant disability or incapacity" means a permanent or significant and substantial disruption of a person's ability to carry out normal life functions.

5.) Medical and scientific judgment should be exercised in deciding whether an adverse event is serious in other situations. Important adverse events that are not immediately life-threatening or do not result in death or hospitalization but may jeopardize the subject or may require intervention to prevent one of the other outcomes listed in the definition above, should also be considered serious. Examples of such events are intensive treatment in an emergency room or at home for allergic bronchospasm, blood dyscrasias, convulsions, drug-induced liver injury or events that necessitate an emergency room visit, outpatient surgery or urgent intervention.

11.2 Recording of AEs and SAEs

11.2.1 Observation period

For the purpose of this study, the period of observation for collection of adverse events extends from the time the patient gives informed consent until the end of the follow-up visit after 12 weeks.

If the investigator detects a Serious Adverse Event in a study patient after the end of the period of observation this should be reported to the Sponsor, particularly if the investigator considers the event possibly related to prior study treatment or procedures. The investigator should contact the sponsor to determine how the adverse event should be documented and reported.

All Adverse Events that occur in the course of a clinical study regardless of the causal relationship must be monitored and followed up until the outcome is known.

11.2.2 Assessment of Seriousness

See Section 11.1.2 Serious Adverse Event for Definition.

11.2.3 Assessment of Severity

The severity (or intensity) of AEs is evaluated according to the grading scale provided in the Cancer Therapy Evaluation Program, Common Terminology Criteria for Adverse Events (CTCAE), v4.03 (please refer to the CTCAE document in the Appendix 8). If an AE term is not listed in the CTCAE classification, the severity of the event should be assessed as follows:

Grade 1 (mild): The adverse event is noticeable to the subject but does not interfere with routine activity.

Grade 2 (moderate): The adverse event interferes with routine activity but responds to symptomatic therapy or rest.

Grade 3 (severe): The adverse event significantly limits the subject's ability to perform routine activities despite symptomatic therapy.

Grade 4 (life-threatening): The subject is at immediate risk of death.

Grade 5 (death): Death related to the adverse event.

To avoid confusion or misunderstanding of the difference between the terms “serious” and “severe”, which are not synonymous, the following note of clarification is provided:

The term "severe" is often used to describe the intensity (severity) of a specific event (as in mild, moderate, or severe myocardial infarction); the event itself, however, may be of relatively minor medical significance (such as severe headache).

This is not the same as "serious," which is based on subject/event outcome or action criteria usually associated with events that pose a threat to a subject's life or functioning. Seriousness (not severity) serves as a guide for defining regulatory reporting obligations.

11.2.4 Assessment of Causality

Careful medical judgment should be exercised to determine if there is a causal relationship between an AE and the investigational product. The following guidance is provided:

The investigator will use medical judgment to determine whether there is evidence for a causal relationship, including all relevant factors such as temporal course and latency, results from de-challenge or re-challenge, pattern of the reaction, known pharmacological properties of the product, and alternative explanations (e.g. other drugs, medical history, concomitant diseases). The investigator will apply the terms “certain”, “probable/likely”, “possible”, “unlikely” or “unrelated” to determine causality. For regulatory E2B reporting, this assessment will be converted to the binary system of “reasonable possibility” and “no reasonable possibility” (see table below). The expression “reasonable possibility”

means to convey in general that there is factual evidence or argument to suggest a causal relationship. The assessment will be documented on the AE and SAE form.

The causality criteria will be used as described in Table 5.

Table 5: Mapping of causality categories to binary causality assessment

<table>
<tr><th>Causality term</th><th>Assessment criteria</th><th>E2B (EudraVigilance Database / EMA)</th></tr>
<tr><td>Certain</td><td>• Event or laboratory test abnormality, with plausible time relationship to drug intake
• Cannot be explained by disease or other drugs
• Response to withdrawal plausible (pharmacologically, pathologically)
• Event definitive pharmacologically or phenomenologically (i.e. an objective and specific medical disorder or a recognized pharmacological phenomenon)
• Re-challenge satisfactory, if necessary</td><td rowspan="3">Reasonable possibility</td></tr>
<tr><td>Probable / Likely</td><td>• Event or laboratory test abnormality, with reasonable time relationship to drug intake
• Unlikely to be attributed to disease or other drugs
• Response to withdrawal clinically reasonable
• Re-challenge not required</td></tr>
<tr><td>Possible</td><td>• Event or laboratory test abnormality, with reasonable time relationship to drug intake
• Could also be explained by disease or other drugs
• Information on drug withdrawal may be lacking or unclear</td></tr>
<tr><td>Unlikely</td><td>• Event or laboratory test abnormality, with a time to drug intake that makes a relationship improbable (but not impossible)
• Disease or other drugs provide plausible explanations</td><td rowspan="2">No reasonable possibility</td></tr>
<tr><td>Unrelated</td><td>There is no evidence or argument to suggest a causal relationship.</td></tr>
</table>

In addition, the causal relationship between an AE and the trabeculectomy (primary surgery/standard of care) and the intravitreal injection will be captured and analyzed.

11.3 Documentation of AEs and SAEs

Adverse events and all information regarding SAEs, whether reported by the subject or observed by the investigator/study personnel, must be documented in the subject's medical record and recorded on the Adverse Event/Serious Adverse Event Forms in the CRF.

One of the aims of the study is to assess the safety and tolerability of the study drug. The investigator is responsible for recording and reporting AEs occurring during the observation period defined in Section 11.2.1.

All AEs occurring during the screening period (i.e., after obtaining the subject's written consent, but before starting study treatment) should be recorded on the Medical History/Current Medical Conditions page in the CRF, except those resulting from a protocol-mandated procedure which should be reported on the Adverse Event/Serious Adverse Event Forms.

Subjects should be routinely followed-up until the End of Study Visit or up to 30 days after the end of study treatment, whichever comes later, for the occurrence of SAEs. The medical monitor can specify a longer period of follow-up to protect the subject's safety.

At any time after the End of Study Visit, if an investigator learns of a SAE that can be reasonably related to study drug, he should promptly notify the sponsor.

11.4 Immediately Reportable Information

11.4.1 Reporting of Serious Adverse Events

All SAEs should be reported immediately, i.e. within 24 hours of learning that the event meets the definition of a SAE. The investigator should complete the Serious Adverse Event Form, assess the causality and send the initial SAE report by fax within 24 hours to the Pharmacovigilance Department.

If the SAE is fatal or life-threatening and is considered by the investigator at least possibly related to the study drug, the Medical Monitor of the sponsor should be informed immediately by telephone and the Pharmacovigilance Department should be informed immediately by fax or email.

The minimum information required for the initial SAE report is:

- Subject number, date of birth
- Description of the event
- Investigational drug information (start date)
- Reporter information
- Causality assessment

Pharmacovigilance Department

spm² - safety projects & more GmbH
Aurum 05
Goldbeckstr. 5
69493 Hirschberg a. d. Bergstraße

Fax: +49 (0) 621 570 – 5971
E-mail: Isarna-PhV@spm2-safety.com

Medical Monitor

M. Feindor, MD
Phone: +49 89 126680-1163
Fax: +49 89 126680-2444
E-mail: Martin.Feindor@synteracthcr.com

Important follow-up information should also be reported to the Pharmacovigilance Department when available. If relevant information is missing at the time of the initial SAE report, the reporter should provide it in follow-up SAE report(s). A follow-up report should contain new, updated or corrected information. The follow-up report should describe whether the event has resolved or continues, if and how it was treated including documentation of all supportive actions taken. For details, refer to the *Guideline for Completion of the Serious Adverse Event Form*.

11.4.2 Other Reportable Information

Certain information, while not necessarily meeting the definition of an AE, is nonetheless reportable.

Reports of medication errors (e.g. overdose/underdose) of study drug with or without symptoms should be handled as SAE. Overdose means that the subject was administered a higher dose than the dose prescribed for the assigned treatment group in this protocol (please refer to Section 7.6 Overdose).

Any pregnancy or fathering of a child, which occurs for up to three months after completion of the study period should be reported as a SAE. Women becoming pregnant during the study will be withdrawn from treatment at the earliest opportunity.

The investigator should immediately report the pregnancy using a Serious Adverse Event Form. It should be clearly stated that no adverse event was observed. In this case, there is no need to complete the “adverse event” page in the case report form. A pregnancy will not be considered as serious as long as there is no serious adverse outcome. In addition, the investigator should provide follow-up information regarding the course of the pregnancy, including perinatal and neonatal outcome, regardless of whether the subject has discontinued participation in the study.

11.5 Reporting of Expeditable Adverse Events to Competent Authorities and Concerned Ethics Committee

11.5.1 SUSAR Reporting

The sponsor will report all serious and unexpected adverse events, which are judged by either the investigator or the sponsor as having a reasonable suspected causal relationship (suspected unexpected serious adverse reaction - SUSAR), to the competent authorities and the concerned ethics committee according to applicable law.

11.5.2 Developmental Safety Update Report (DSUR)

The sponsor will prepare and submit a DSUR annually to competent authorities and concerned ethics committees.

12 STATISTICAL ANALYSIS

12.1 Statistical Methods

Tables, raw data listings and figures (TLFs) will be generated using the software SAS© Version 9 or higher.

A separate Statistical Analysis Plan (SAP) will be finalized that details the planned statistical analysis and may include necessary adaptations to the planned statistical analysis before database closure. Any deviations from the analysis preplanned in the protocol or the SAP will be described and justified in the final study report.

12.1.1 Statistical Hypothesis

No statistical hypothesis is generated, as this is a phase I study without confirmatory statistical testing and formal sample size estimation.

12.1.2 Level of Significance, Multiple Comparisons and Multiplicity

No significance tests will be performed and hence no level of significance has to be defined.

12.1.3 Determination of Sample Size

Up to four dose levels with a cohort size of 3 subjects, i.e. a total of up to 12 subjects are planned in total excluding replacement subjects. The sample size is not based on any statistical rationale but considered large enough for making relevant clinical evaluations and conclusions.

12.2 Planned Analysis

Primary and secondary variables will be evaluated exploratory. All relevant data on subjects (CRF data, laboratory data) will be analyzed descriptively grouped by dose cohorts and visits.

Individual subject data will be presented in listings (sorted by dose cohort, subject number and visit if applicable). All data collected in the CRF and included in the database will be listed.

All data obtained in this study and documented in the Case Report Forms or derived from external data from source documents uploaded into eCRF (e.g. Electroretinogram) will be listed and selected data will be summarized with statistics or frequency tables as appropriate.

12.2.1 Demographic Data and Other Baseline Characteristics

Demographics and other baseline characteristics will be summarized in total and by dose cohort by means of summary statistics (number of subjects, mean, standard deviation, minimum, median, maximum) for continuous variables and by absolute and relative frequencies for categorical variables. Baseline characteristics are defined as all results of the examinations performed prior to the IMP administration.

12.2.2 Planned Analysis for Primary Endpoint(s)

The primary endpoint for safety and tolerability is the type and frequency of adverse events.

Coding: All AEs reported in this study will be coded using the Medical Dictionary for Regulatory Activities (MedDRA), current version 17.1 or higher.

All AEs occurring during the course of the clinical trial will be classified into:

- **pre-treatment adverse events** AEs with onset prior to first administration of IMP
- treatment-emergent AEs (**TEAE):** AEs with onset at or after first administration of IMP. AEs with missing onset date will be classified as treatment-emergent as well.
- study drug-related TEAEs (**ADRs**): TEAEs that are reported as "*Certain*", "*Probable/likely*" or "*Possible*" related to study medication will be considered treatment-related; missing classifications concerning study drug relationship will also be considered treatment-related.

An overview AE summary table will be prepared showing the number and percentage of subjects with at least one event and the total number of events for the following selections:

- pre-treatment AEs
- TEAEs
- study drug-related TEAEs (ADRs)
- TEAEs related to intravitreal injection (injection site reactions)
- TEAEs related to primary surgery
- serious TEAEs
- study drug-related serious TEAEs (serious ADRs)

In addition, frequency tables will be prepared by MedDRA terms (system organ class [SOC], preferred term [PT]) showing the following:

- All TEAEs
- TEAEs by CTCAE severity grading
- TEAEs by causal relationship to study drug, to intravitreal injection and to primary surgery
- Study-drug related TEAEs (ADRs)
- Study-drug related TEAEs (ADRs) by CTCAE severity grading
- Serious TEAEs

The analysis of AEs will include summary tables displaying counts and percentages of subjects experiencing adverse events by system organ class and preferred term. If a subject has more than one AE which codes to the same preferred term, the subject will be counted only once for that preferred term. The total number of events documented per SOC and PT will also be displayed.

12.2.3 Planned Analysis for Secondary Endpoint(s)

All main secondary endpoints are summarized in section 4.1.2. Depending on the outcome of data (continuous, categorical or dichotomous), appropriate summary statistics or frequency count tables are prepared showing each parameter classified by dose cohort and visit.

For continuous outcome variables (e.g. IOP) absolute changes from baseline (Day -1) will be calculated.

12.2.4 Further variables

Clinical laboratory test results, physical examination and vital signs parameters will be summarized by time point with descriptive statistics.

Clinical laboratory test results will be marked whether the result is below, within or above the respective reference range. The number of values below, within and outside the reference range will be counted.

Results of pregnancy tests, medical history and concomitant medication will be listed only.

12.3 Statistical Criteria for Study Termination

No criteria for early termination of the study from a statistical perspective are considered as no sequential interim analysis is performed.

12.4 Handling of Missing and Unused Spurious Data

Only non-missing data will be analyzed, no missing value replacement procedure will be deployed for clinical data.

12.5 Definition of Study Populations

The statistical analysis will be based on the following study populations:

All subjects who received at least one dose of the assigned study medication will be included in the safety evaluation (safety analysis set = SAF).

All subjects included into the SAF and who attend at least study visit "Follow Up (FU)" (6 weeks post injection) constitute the full analysis set (FAS).

A per-protocol analysis set (PPS) will not be defined due to the exploratory character of the study. Nevertheless, during a data review meeting at end of the study, protocol deviations will be determined and classified whether they are to be considered as major or minor deviations.

12.6 Interim Analysis

No formal interim analysis with the purpose of adapting the study design, adjusting the sample size or early stopping is planned for this study.

Interim data packages are however prepared for the CRCs.

12.7 Subgroup analysis

A subgroup analysis is not planned. However, if there is evidence that the study population is inhomogeneous and that this inhomogeneity could possibly influence the statistical analysis, descriptive, explorative subgroup analyses can be done.

13 QUALITY CONTROL AND QUALITY ASSURANCE

13.1 Data Recording

All CRF data will be collected using an eCRF within a fully validated and CFR 21 Part 11 compliant Electronic Data Capture (EDC) system. All data will be entered into the CRF by the Site Staff. These data will then be source data verified and reviewed by the study monitor before data cleaning by Data Management is performed. All queries will be raised and resolved within the EDC system. During entry programmatic checking of the data will be performed and once saved into the database more complex programmatic checks will also be performed. During the conduct of the study all system users will have real time access to the data, the level of access to the data and study privileges will be determined by their user role.

After all queries have been resolved, the SAP approved and signed, and any summary/analysis populations approved, the database will be locked and the data released for summary and analysis. All summary and analysis of the data will be performed using SAS® version 9.1 or later.

13.2 Monitoring

An independent monitor or other authorized personnel will be allowed to inspect the various records of the trial on request (CRFs and other pertinent data), provided that subject confidentiality is maintained. Monitoring will be conducted in accordance with applicable regulations, GCP and the sponsor representative's procedures. It is the monitor's responsibility to inspect the case report forms at regular intervals throughout the trial to verify adherence to the protocol, the completeness, accuracy and consistency of the data, and adherence to ICH GCP guidelines. The monitor should have access to subject charts, laboratory reports and other subject records needed to verify the entries on the CRFs.

During the study, the Investigator must make study data accessible to the study monitors, the Sponsor (or a third party auditor assigned by the Sponsor), and relevant ECs and regulatory agencies. A file for each subject must be maintained that includes the signed informed consent form and all source documentation related to that subject. The Investigator must ensure the availability of source documents from which the information in the CRF was derived.

Clinical documentation relevant to the study includes all records in any form (including, but not limited to, written, electronic, magnetic, and optical records, and scans, X-rays and electrocardiograms) that describe or record the methods, conduct and/or results of the study, the factors affecting the study and the actions taken. Source data is all information in original records and certified copies of original records of clinical findings, observation, or other activities in a clinical study necessary for the reconstruction and evaluation of the study. Source data are contained in source documents which comprise clinical documentation, data and records (e.g. hospital records, clinical and office charts, laboratory notes, memoranda, subject diaries or evaluation checklists, pharmacy dispensing records, recorded data from automated instruments and data and records arising from other departments such as the pharmacy, laboratory and medico-technical departments).

13.3 Audits and Inspections

The Sponsor or Sponsor representative may at any time during or after completion of the study conduct a GCP audit. Prior notice will be given to each site selected for audit. The investigator agrees to allow the auditor direct access to all relevant documents and to allocate his/her time and the time of his/her staff to the auditor to discuss findings and any relevant issues.

Regulatory agencies may conduct regulatory inspections of this study. If a regulatory authority requests an inspection, the investigator must inform the sponsor or the Sponsor’s representative immediately about this request. The investigator agrees to allow the inspector(s) direct access to all relevant documents and to allocate his/her time and the time of his/her staff to the auditor to discuss findings and any relevant issues.

13.4 Data Retention and Availability

The Investigator must retain a comprehensive and centralized filing system of all trial-related documentation that is suitable for inspection by the sponsor and representatives of regulatory authorities.

The Investigator must arrange for the retention of the subject identification codes for at least 15 years after the completion or discontinuation of the trial. Subject files and other source data (including copies of protocols, case report forms, original reports of test results, agent-dispensing logs, correspondence, records of informed consent, and other documents pertaining to the conduct of the trial) must be kept for the maximum period of time permitted by the institution.

No trial document will be destroyed without prior written agreement between the sponsor and the Investigator. Should the Investigator wish to assign the trial records to another party or move them to another location, written agreement must be obtained from Isarna Therapeutics.

14 ETHICS REVIEW/INFORMED CONSENT

The Investigator will ensure that this study is conducted in full conformity with the current version of the Declaration of Helsinki, with the ICH GCP Guidelines and with the local drug law, whichever affords the greater protection to the subject.

The final study protocol and subject informed consent form will be approved by the appropriate Ethics Committee (EC) for each investigational site. Approval will be received in writing before initiation of the study.

Changes to the protocol during the trial will be documented as amendments. Depending on the contents of the amendment and local legal requirements, the amendment will be submitted for approval to the

relevant ECs and to the relevant competent authorities (CAs) prior to implementation. Exceptions are cases of changes made to protect subject safety, which will be implemented immediately.

Proposed amendments of Isarna Therapeutics sponsored protocols must be submitted to Isarna Therapeutics for review and approval, and then to the EC. Amendments may be implemented only after a copy of the EC's approval letter has been transmitted to the sponsor. Amendments that are intended to eliminate an apparent immediate hazard to subjects may be implemented prior to receiving EC approval. However, in this case, approval must be obtained as soon as possible after implementation.

If an amendment substantially alters the trial design, increases the potential risk to the subjects, affects the treatment of the subject or might otherwise influence the willingness of the subject to participate in the trial, then the information sheet must be revised and submitted to the relevant EC and, where necessary, to the relevant CAs, for review and approval. Where a subject is currently undergoing trial procedures and is affected by the amendment, the subject must be asked to consent again using the new information sheet.

The Investigator will be responsible for assuring that continuing review (at least once per year) of the study is performed by the EC throughout the duration of the study. This process may be facilitated by the Sponsor. Where directly issued by the Investigator, copies of these reports and of the notice of approval must be sent to the Sponsor.

14.1 Ethical Conduct of the Study

The study will be conducted in accordance with ICH GCP, the Declaration of Helsinki, the EU Clinical Trials Directive 2001/20/EC, the GCP Directive 2005/28/EC, and the requirements of local ECs.

14.2 Informed Consent

It is the Investigator's responsibility to obtain written informed consent from the subject after adequate explanation of the aims, methods, anticipated benefits, and potential hazards of the study and before entry into the study. The subject should be given a copy of the signed and dated informed consent documentation. The original signed and dated copy of the informed consent must be retained in the institution's records, and is subject to inspection by representatives of the Sponsor, or representatives from regulatory agencies.

14.3 Subject Participation Card

A study participation card will be provided to each subject on the trial. The card will indicate that they are participating in a clinical trial, and give the name and contact details of the Investigator/study site. The subject will be asked to retain this card while they are participating in the trial and show it to any other medical practitioners they consult during this time. They will be advised to contact the Investigator/study site if there are any questions.

14.4 Insurance and Finance

Insurance for the subjects participating in this trial will be arranged by Isarna Therapeutics, as Sponsor of the clinical trial, in accordance with the regulatory requirements of the countries involved. A copy of the country-specific insurance certificates will be held in the trial master file (TMF) and in the Investigator site file.

Financial issues will be agreed upon by a separate contract document. For each participating subject the Sponsor has taken out an insurance contract covering the amount requested by the respective national laws. All participating subjects will be informed about the existence of the insurance policy in detail in the subject informed consent. They have the right to review the terms and conditions described therein.

14.5 Pre-Study Documentation Requirements

The following documents may be required before an investigational agent can be shipped from the

Sponsor or the manufacturer:

- signed protocol;
- copy of approved informed consent form;
- copy of the EC approval of the protocol and consent form;
- curricula vitae of the Investigators;
- name and address of the EC and membership of the EC;
- laboratory normal ranges and documentation of laboratory certification;
- local regulatory approval and approval to import the investigational medicinal products (if required);
- agreement to destroy unused investigational medicinal products supplies at the site according to local written procedures, if applicable; and
- signed letter of (financial) agreement, if applicable.

14.6 Confidentiality of Subject Data

Permission for direct access to subject’s data will be sought in writing by the Investigator and from the subject as part of the informed consent procedure. This gives permission to examine, analyze, verify and reproduce any records and reports that are important to the evaluation of the study. Any party (e.g., domestic and foreign regulatory authorities, monitors and auditors) with direct access must take all reasonable precautions within the constraints of the applicable regulatory requirement(s) to maintain the confidentiality of the subject’s identities and Sponsor’s proprietary information.

It is the monitor’s responsibility to verify that each subject has consented, in writing, to direct access.

It is to be ensured by the Investigator that documents given to the sponsor or its representatives do not contain the name or address of the subject, or other information that would affect the anonymity of the subject as stipulated by the local legal requirements.

14.7 Premature Study Termination

The study may be prematurely terminated (by the EC, CA or the sponsor) if the perception of the benefit/risk becomes unfavorable for the continuation of the study.

If the study is prematurely terminated or suspended for any reason, the Investigator should promptly inform the subjects, should assure appropriate therapy and follow-up for the subjects and the institution where the study was being performed.

15 DISCLOSURE OF INFORMATION

All data and records provided by Isarna or generated during the study (other than a subject’s medical records) and all data and inventions discovered in the course of conducting the study, whether patentable or not, are the sole and exclusive property of Isarna.

The investigator and other study site personnel will keep strictly confidential any information provided by Isarna related to this study and all data and records generated in the course of conducting the study. They will not use the information, data or records for any other purpose than conducting the study.

The investigator(s) and the institution may use the scientific data generated during this study for their own non-commercial research, but only within the group of people underlying regulations of confidentiality and disclosure of information of this protocol. The investigator(s) may use the scientific data generated during this study for scientific publications of any kind, after consultation of the Sponsor regarding Intellectual Property. The investigator(s) will allow Isarna to review the proposed publication or disclosure in due time before submission for publication, presenting, using for instructional purposes or otherwise disclosing the results of the study. In case the proposed publication or disclosure will risk Isarna’s ability to patent any invention related to the study, the publication or disclosure will be modified or delayed to allow Isarna to file a patent application.

The first publication (of a full text report) of the outcome of the study will be a complete, joint multicenter publication. Authorship will be discussed with all parties involved, and depend on factors as recruitment and participation in protocol development. Publication or disclosure of partial results from an individual study site will generally not be allowed until after the publication of a first full text report of the multicenter effort.

Isarna will prepare an integrated clinical study report in accordance with the ICH Harmonized Tripartite Guideline (E3 - Structure and Content of Clinical Study Reports) after complete evaluation of the study results. The coordinating investigators will also sign the clinical study report.

16 REFERENCES

16.1 Source Documents

Investigator's Brochure ISTH0036 version 4.0 dated 31 Aug 2016

16.2 Literature

Ali, M., McKibbin, M., Booth, A., al., E., 2009. Null Mutations in LTBP2 Cause Primary Congenital Glaucoma. Am J Hum Genet. 84**,** 664-71.

Allen, J.B., Davidson, M.G., Nasisse, M.P., al., E., 1998. The lens influences aqueous humor levels of transforming growth factor-beta 2. Graefes Arch Clin Exp Ophthalmol. 236**,** 305-11.

Bettin, P., Di Matteo, F., 2013. Glaucoma: present challenges and future trends. Ophthalmic Res. 50**,** 197-208.

Bollinger, K.E., Crabb, J.S., Yuan, X., Putliwala, T., Clark, A.F., Crabb, J.W., 2011. Quantitative Proteomics: TGF{beta}2 Signaling in Trabecular Meshwork Cells. Invest Ophthalmol Vis Sci. 52**,** 8287-94.

Border, W.A., Noble, N.A., 1994. Transforming growth factor beta in tissue fibrosis. N Engl J Med. 331**,** 1286-92.

Congdon, N., Wang, F., Tielsch, J.M., 1992. Issues in the epidemiology and population-based screening of primary angle-closure glaucoma. Surv Ophthalmol. 36**,** 411-23.

Connor, T.B., Jr., Roberts, A.B., Sporn, M.B., Danielpour, D., Dart, L.L., Michels, R.G., de Bustros, S., Enger, C., Kato, H., Lansing, M., et al., 1989. Correlation of fibrosis and transforming growth factor-beta type 2 levels in the eye. J Clin Invest. 83**,** 1661-6.

Cordeiro, M.F., Mead, A., Ali, R.R., Alexander, R.A., Murray, S., Chen, C., York-Defalco, C., Dean, N.M., Schultz, G.S., Khaw, P.T., 2003. Novel antisense oligonucleotides targeting TGF-beta inhibit in vivo scarring and improve surgical outcome. Gene Ther. 10**,** 59-71.

Damiano, V., Caputo, R., Garofalo, S., Bianco, R., Rosa, R., Merola, G., Gelardi, T., Racioppi, L., Fontanini, G., De Placido, S., Kandimalla, E.R., Agrawal, S., Ciardiello, F., Tortora, G., 2007. TLR9 agonist acts by different mechanisms synergizing with bevacizumab in sensitive and cetuximab-resistant colon cancer xenografts. Proc Natl Acad Sci U S A. 104**,** 12468-73.

Danesh-Meyer, H.V., Ku, J.Y., Papchenko, T.L., al., E., 2006. Regional correlation of structure and function in glaucoma, using the Disc Damage Likelihood Scale, Heidelberg Retina Tomograph, and visual fields. Ophthalmology. 113**,** 603-11.

Engler, C., Chakravarti, S., Doyle, J., Eberhart, C.G., Meng, H., Stark, W.J., Kelliher, C., Jun, A.S., 2011. Transforming growth factor-beta signaling pathway activation in Keratoconus. Am J Ophthalmol. 151**,** 752-759 e2.

Freedman, J., Iserovich, P., 2013. Pro-Inflammatory Cytokines in Glaucomatous Aqueous and Encysted Molteno Implant Blebs and Their Relationship to Pressure. Invest Ophthalmol Vis Sci. 54**,** 4851–4855.

Fuchshofer, R., Welge-Luessen, U., Lütjen-Drecoll, E., 2003. The effect of TGF-beta2 on human trabecular meshwork extracellular proteolytic system. Exp Eye Res. 77**,** 757-65.

Fuchshofer, R., Yu, A.H., Welge-Lüssen, U., al., E., 2007. Bone morphogenetic protein-7 is an antagonist of transforming growth factor-beta2 in human trabecular meshwork cells. Invest Ophthalmol Vis Sci. 48**,** 715-26.

Fuchshofer, R., 2011. The pathogenic role of transforming growth factor-beta2 in glaucomatous damage to the optic nerve head. Exp Eye Res. 347**,** 279-90.

Fuchshofer, R., Tamm, E.R., 2012. The role of TGF-beta in the pathogenesis of primary open-angle glaucoma. Cell Tissue Res. 347**,** 279-90.

Georgoulas, S., Dahlmann-Noor, A., Brocchini, S., Khaw, P.T., 2008. Modulation of wound healing during and after glaucoma surgery. Prog Brain Res. 173**,** 237-54.

Gordon-Thomson, C., De Longh, R.U., Hales, A.M., al., E., 1998. Differential cataractogenic potency of TGF-beta1, -beta2, and -beta3 and their expression in the postnatal rat eye. Invest Ophthalmol Vis Sci. 39**,** 1399-409.

Granstein, R.D., Staszewski, R., Knisely, T.L., 1990. Aqueous humor contains transforming growth factor-beta and a small (less than 3500 daltons) inhibitor of thymocyte proliferation. J Immunol. 144**,** 3021-7.

Helbig, H., Kittredge, K.L., Coca-Prados, M., al., E., 1991. Mammalian ciliary-body epithelial cells in culture produce transforming growth factor-beta. Graefes Arch Clin Exp Ophthalmol. 229**,** 84-7.

Ihn, H., 2002. Pathogenesis of fibrosis: role of TGF-beta and CTGF. Curr Opin Rheumatol. . 14**,** 681-5.

Inatani, M., Tanihara, H., Katsuta, H., Honjo, M., Kido, N., Honda, Y., 2001. Transforming growth factor-beta 2 levels in aqueous humor of glaucomatous eyes. Graefes Arch Clin Exp Ophthalmol. 239**,** 109-13.

ISRN-PRD-1443. ASPH_0036 - Toxicology study in the Dutch-Belted rabbit after 3 intravitreal injections at 2-week intervals following 12-week recovery period. Final Report. 20150731.

ISRN-PRD-1457a. ASPH_0036 – Preliminary local tolerance, tissue distribution and pharmacodynamic study after a single intravitreal injection in the Beagle dog. Final Report. 20150727.

ISRN-PRD-1501. ISTH0036 - 9-month toxicity study in the Dutch Belted rabbit, including an interim sacrifice (Week 18), by the intravitreal route, followed by a 12-week observation period. Final Report. 20160721.

ISRN-PRD-1502. ISTH0036 Exploratory Safety and Toxicokinetic Study in the Cynomolgus Monkey with a 12-Week Treatment-Free Period. Final Report. 160728.

Jampel, H.D., Roche, N.S., Stark, W.J., al., E., 1990. Transforming growth factor-beta in human aqueous humor. Curr Eye Res. 9**,** 963-9.

Kon, C.H., Occleston, N.L., Aylward, G.W., Khaw, P.T., 1999. Expression of vitreous cytokines in proliferative vitreoretinopathy: a prospective study. Invest Ophthalmol Vis Sci. 40**,** 705-12.

Kria, L., Ohira, A., Amemiya, T., 1996. Immunohistochemical localization of basic fibroblast growth factor, platelet derived growth factor, transforming growth factor-beta and tumor necrosis factor-alpha in the pterygium. Acta Histochem. 98**,** 195-201.

Lockwood, A., Brocchini, S., Khaw, P.T., 2013. New developments in the pharmacological modulation of wound healing after glaucoma filtration surgery. Curr Opin Pharmacol. 13**,** 65-71.

Min, S.H., Lee, T.I., Chung, Y.S., al., E., 2006. Transforming growth factor-beta levels in human aqueous humor of glaucomatous, diabetic and uveitic eyes. Korean J Ophthalmol. 20**,** 162-5.

Ochiai, Y., Ochiai, H., 2002. Higher concentration of transforming growth factor-beta in aqueous humor of glaucomatous eyes and diabetic eyes. Jpn J Ophthalmol. 46**,** 249-53.

Ozcan, A.A., Odzdemir, N., Canataroglu, A., 2004. The aqueous levels of TGF-β2 in patients with glaucoma. International Ophthalmology. 25**,** 19-22.

Park, H.Y., Kim, J.H., Park, C.K., 2013. VEGF induces TGF-beta1 expression and myofibroblast transformation after glaucoma surgery. Am J Pathol. 182**,** 2147-54.

Pasquale, L.R., Dorman-Pease, M.E., Lutty, G.A., 1993. Immunolocalization of TGF-beta 1, TGF-beta 2, and TGF-beta 3 in the anterior segment of the human eye. Invest Ophthalmol Vis Sci. 34**,** 23-30.

Pena, J.D., Taylor, A.W., Ricard, C.S., Vidal, I., Hernandez, M.R., 1999. Transforming growth factor beta isoforms in human optic nerve heads. Br J Ophthalmol. 83**,** 209-18.

Pfeffer, B.A., Flanders, K.C., Guérin, C.J., al., E., 1994. Transforming growth factor beta 2 is the predominant isoform in the neural retina, retinal pigment epithelium-choroid and vitreous of the monkey eye. Exp Eye Res. 59**,** 323-33.

Phanish, M.K., Winn, S.K., Dockrell, M.E., 2009. Connective tissue growth factor-(CTGF, CCN2)--a marker, mediator and therapeutic target for renal fibrosis. Nephron Exp Nephrol. 114**,** 83-92.

Picht, G., Grehn, F., 1998. Classification of filtering blebs in trabeculectomy: biomicroscopy and functionality. Curr Opin Ophthalmol. 9**,** 2-8.

Picht, G., Welge-Luessen, U., Grehn, F., Lutjen-Drecoll, E., 2001. Transforming growth factor beta 2 levels in the aqueous humor in different types of glaucoma and the relation to filtering bleb development. Graefes Arch Clin Exp Ophthalmol. 239**,** 199-207.

Prendes, M.A., Harris, A., Wirostko, B.M., Gerber, A.L., Siesky, B., 2013. The role of transforming growth factor beta in glaucoma and the therapeutic implications. Br J Ophthalmol. 97**,** 680-6.

Quigley, H.A., Broman, A.T., 2006. The number of people with glaucoma worldwide in 2010 and 2020. Br J Ophthalmol. 90**,** 262-7.

Quigley, H.A., 2011. Glaucoma. Lancet. 377**,** 1367-77.

Rohen, J.W., Witmer, R., 1972. Electron microscopic studies on the trabecular meshwork in glaucoma simplex. Graefes Arch Clin Exp Ophthalmol. 183**,** 251-66.

Saika, S., 2006. TGFbeta pathobiology in the eye. Lab Invest. 86**,** 106-15.

Sambhara, D., Aref, A.A., 2014. Glaucoma management: relative value and place in therapy of available drug treatments. Ther Adv Chronic Dis. 5**,** 30-43.

Schlotzer-Schrehardt, U., Zenkel, M., Kuchle, M., Sakai, L.Y., Naumann, G.O., 2001. Role of transforming growth factor-beta1 and its latent form binding protein in pseudoexfoliation syndrome. Exp Eye Res. 73**,** 765-80.

Stein, H., Hausen, P., 1969. Enzyme from calf thymus degrading the RNA moiety of DNA-RNA Hybrids: effect on DNA-dependent RNA polymerase. Science. 166**,** 393-5.

Tektas, O.Y., Lütjen-Drecoll, E., 2009. Structural changes of the trabecular meshwork in different kinds of glaucoma. Exp Eye Res. 88**,** 763-75.

Tomarev, S.I., Wistow, G., Raymond, V., al., E., 2003. Gene expression profile of the human trabecular meshwork: NEIBank sequence tag analysis. Invest Ophthalmol Vis Sci. 44**,** 2588-96.

Tovar-Vidales, T., Clark, A.F., Wordinger, R.J., 2011. Transforming growth factor-beta2 utilizes the canonical Smad-signaling pathway to regulate tissue transglutaminase expression in human trabecular meshwork cells. Exp Eye Res. 93**,** 442-51.

Tripathi, R.C., Li, J., Chan, W.F., Tripathi, B.J., 1994. Aqueous humor in glaucomatous eyes contains an increased level of TGF-beta 2. Exp Eye Res. 59**,** 723-7.

Trivedi, R.H., Nutaitis, M., Vroman, D., Crosson, C.E., 2011. Influence of race and age on aqueous humor levels of transforming growth factor-beta 2 in glaucomatous and nonglaucomatous eyes. J Ocul Pharmacol Ther. 27**,** 477-80.

Van Bergen, T., Van de Velde, S., Vandewalle, E., Moons, L., Stalmans, I., 2014. Improving patient outcomes following glaucoma surgery: state of the art and future perspectives. Clin Ophthalmol. 8**,** 857-67.

Wallentin, N., Wickström, K., Lundberg, C., 1998. Effect of cataract surgery on aqueous TGF-beta and lens epithelial cell proliferation. Invest Ophthalmol Vis Sci. 39**,** 1410-8.

Wormstone, I.M., 2002. Posterior capsule opacification: a cell biological perspective. Exp Eye Res. 74**,** 337-47.

Yamamoto, N., Itonaga, K., Marunouchi, T., Majima, K., 2005. Concentration of transforming growth factor beta2 in aqueous humor. Ophthalmic Res. 37**,** 29-33.

Zode, G.S., Sethi, A., Brun-Zinkernagel, A.M., Chang, I.F., Clark, A.F., Wordinger, R.J., 2011. Transforming growth factor-beta2 increases extracellular matrix proteins in optic nerve head cells via activation of the Smad signaling pathway. Mol Vis. 17**,** 1745-58.

17 APPENDICES

Appendix 1 Study Flow Charts

Table 6: Study Flow Chart

			**Core Study**					**Post-Study Follow-up**		
	**Screening**	Wash-out period according to trabeculectomy standard procedure	**Hospitalization**			**FU**	**EOS**	**PSFU**		
**Weeks**						**W6**	**W12**	**M6**	**M12**	**1 Year post EOS**
**Study Day**	**Day -42 to Day -2**		**Day -1 Baseline**	**Day 1**	**Day 3[1]**	**Day 43+2**	**D85±2**			
**Study Visit**	**1**		**2**			**FU**	**EOS**	**PSFU1**	**PSFU2**	**PSFU3**
Written informed consent	X									
Demographics, height, weight	X									
Medical history/current medical conditions (incl. medication)	X									
Check of inclusion / exclusion criteria	X									
Pregnancy test for women of child-bearing potential	X[2]		X[3]							
Safety laboratory[4] (hematology, biochemistry, coagulation) and urinalysis[5]	X		X			X	X			
Physical examination	X					X	X			
Vital signs[6]	X		X	X[7]	X	X	X			
Trabeculectomy (as standard of care)				X						
Injection of ISTH0036[8]				X[9]						
Visual acuity	X		X		X	X	X			
Visual field	X		X			X	X			
Slit-lamp biomicroscopy	X		X			X	X			
Bleb filtering evaluation					X	X	X			
Intraocular pressure	X		X			X	X			

Heidelberg Retina tomograph II (HRTII)	X					X	X			
Optic disc photographs	X					X	X			
Electroretinogram	X					X				
Documentation of interventions				X	X	X	X			
Adverse events			X	X	X	X	X			
Concomitant medication			X	X	X	X	X			
Additional written informed consent								X		
Concomitant anti-glaucoma medication								X	X	
Long term IOP efficacy assessment								X[10]	X[10]	
Long term safety assessment								X	X	X[11]

[1] During hospitalization the subject will receive standard hospital care.
[2] Serum pregnancy test to be performed within 7 days prior to study treatment.
[3] To confirm the absence of pregnancy a urine test may be used.
[4] Hematology: Hemoglobin, hematocrit, red blood cell count (RBC), white blood cell count (WBC) with differential WBC, and platelet count; biochemistry: creatinine, LDH, calcium, electrolytes (sodium and potassium), total bilirubin, GGT, albumin, alkaline phosphatase, ASAT, ALAT, and glucose; coagulation: INR (international normalized ratio).
[5] Urinalysis: Presence of glucose, protein and blood in urine will be assessed by dipstick.
[6] Vital signs: Heart rate, systolic and diastolic blood pressure (sitting), body temperature.
[7] To be done before surgery and 2h and 4h post-op.
[8] The study site standard procedure for intravitreal injection will be used.
[9] Injection of ISTH0036 intraoperatively at the end of the trabeculectomy surgical procedure (post MMC).
[10] Collection of IOP follow-up assessment data standardly performed either at trial site or at patient's ophthalmologist and reported to trial site
[11] Patient contacted by phone.

Appendix 2 Cohort Review Committee Process

A formal Cohort Review Committee (CRC) is installed for this First-in-Human study and will be responsible for safeguarding the interests of study patients, and assessing the safety and tolerability of the study treatment in an ongoing fashion. This Appendix will define the primary responsibilities of the CRC, its membership, and the purpose and timing of its meetings.

Role of the CRC

The CRC evaluates study data on an ongoing basis to assure patients safety in the First-in-Human study ISTH-01-111. The CRC will be the main platform for communication and discussion of all clinical information to ensure that new safety findings and all other relevant information are transmitted to all participating sites and that the integrity of the study design is not compromised. The committee monitors data and makes recommendations based on the data that are periodically reviewed.

Primary Responsibilities of the CRC

- Initially, familiarize themselves with the research protocol, especially the safety reporting section, informed consent and other relevant document(s).
- Ongoing regularly scheduled evaluation of the progress of the trial, which may include some or all of the following:
    - Monitoring adverse events, dose limiting toxicities, patient dropouts, compliance, or any complaints
    - Monitoring data quality for completeness, timeliness, and accuracy
    - Monitoring patient recruitment, accrual, and retention
    - Looking at risks vs. benefits
    - Reviewing any other factors that might affect study outcome
- Consider new safety information that may have an impact on the safety of the participants or the ethics of the trial
- Consider factors external to the study when relevant information becomes available, such as scientific or therapeutic developments that may have an impact on the safety of the participants or the ethics of the trial.
- Ensure confidentiality of trial data and results of monitoring
- Make decisions on:
    - Dose limiting toxicities
    - Dose escalation and dose schedules and any other relevant dosing decision
    - Any protocol amendments that occurred between review periods
    - Study suspension, termination, or protocol amendments based on review of safety data or interim analysis, where applicable, in case of significant safety events.

Membership of the CRC

The main responsibility of the CRC is the review of dose limiting toxicities and other significant adverse events and the decision on dose escalation. It is therefore essential to organize the review of emerging safety data by the investigators, together with the sponsor's representatives. Accordingly the CRC will consist of principal investigators or delegates of all participating centers, the medical monitor, the drug safety officer and other team members from the sponsor as voting or non-voting members. The roles are defined below. In addition independent experts may be invited/consulted where appropriate.

The CRC will consist of at least three (3) voting members and at least one representative of each center and sponsor attending the meetings or conference calls.

Voting Attendees:

- Coordinating Investigator (LKP)
- Principal Investigators of each participating centers or a delegate thereof

- Medical Monitor

Non-Voting Attendees:

- CRO Representatives
- Sponsors Representatives
- Biostatistics as needed (statistician may be responsible for preparing data for the CRC meetings)
- Other, i.e. external expert

CRC Procedures

1. The coordinating investigator (LKP according to German drug law) will be the CRC chair.
2. Preparation of the meetings will be coordinated by the project manager of the CRO or the sponsor
3. The first CRC meeting will take place after the first 3 patients in cohort 1 have completed their DLT period.
4. CRC meetings will generally be held after completion of each cohort (i.e. dose level), after occurrence of a dose limiting toxicity or any other emerging clinically significant event. The CRC will review accumulated data on safety (including any SAEs).
5. The CRC chair may call an emergency meeting at any time should questions of patient safety arise.
6. The clinical project manager will function as an interface between all parties to ensure that all relevant safety information of each patient is distributed to the study team members.
7. All materials, discussions, and proceedings of the CRC are completely confidential. Members and other participants present at CRC meetings are expected to maintain confidentiality.

CRC Meeting Materials

It is responsibility of the principal investigator to have adequately documented treatment data and case narrative available for monitoring and data collection in preparation of a CRC meeting. Data listings are extracted from the eCRF database and distributed to all CRC members.

Reports contain aggregate safety data which may be separated by cohort, or other data or formats as requested by the CRC.

CRC Meeting Minutes

For all decisions a consensus vote should be achieved. Decisions made by the CRC will be laid down meeting minutes prepared by the project manager. Minutes will be distributed to all CRC members for approval. Any concerns will be brought to the attention of the CRC chair who will make any recommendations if appropriate. In case of disagreement of one CRC member not having participated in the CRC meeting, an emergency meeting should be scheduled to reach a consensus.

Once approved by the CRC, the minutes will be sent to all study team members together with a Dose escalation approval form.

Appendix 3 Slit-lamp biomicroscopy evaluations

Both eyes will be examined.

	Grading
**Lid erythema**	0 = none (normal)<br>1 = mild (redness localized to a small region of the lid(s) margin OR skin)<br>2 = moderate (redness of most or all lid margin OR skin)<br>3 = severe (redness of most or all lid margin AND skin)<br>4 = very severe (marked diffuse redness of both lid margins AND skin)
**Lid edema**	0 = none (normal)<br>1 = mild (localized to a small region of the lid)<br>2 = moderate (diffuse, most or all lid but not prominent/protruding)<br>3 = severe (diffuse, most or all lid AND prominent/protruding)<br>4 = very severe (diffuse AND prominent/protruding AND reversion of the lid)
**Conjunctival erythema**<br>Redness of the eye will be graded by ORA Redness Scale (Grade 0-4)	0 = none (normal)<br>1 = mild (a flush reddish color predominantly confined to the palbebral or bulbar conjunctiva)<br>2 = moderate (more prominent red color of the palbebral or bulbar conjunctiva)<br>3 = severe (definite redness of palbebral or bulbar conjunctiva)<br>4 = very severe (severe redness of palbebral and bulbar conjunctiva)
**Conjunctival edema**	0 = none (normal)<br>1 = mild (slight localized swelling)<br>2 = moderate (moderate/medium localized swelling or mild diffuse swelling)<br>3 = severe (severe diffuse swelling)<br>4 = very severe (very prominent/protruding diffuse swelling)
**Epithelial Defects**<br>Epithelial defects will be evaluated by Fluorescein corneal staining and will be graded by the Modified Oxford Scale (Grade 0-5).	0 = no staining dots<br>0.5 = 1 to 3 staining dots<br>1 = mild<br>2 = moderate<br>3 = between moderate and severe

	4 = severe<br>5 = very severe
**Anterior Chamber Depth** (Slit beam = 0.3 mm wide, 1.0 mm long)	Normal<br>Other (specify)
**Cells**	Normal<br>Trace (0-5 cells)<br>Mild (6-10 cells)<br>Moderate (11-20 cells)<br>Marked (21-50 cells)<br>Severe (>50 cells)
**Flare**	None<br>Mild<br>Moderate<br>Marked<br>Severe
**Epithelium**	Normal<br>Punctate staining<br>Other (specify)<br>Precipitates (specify)
**Stroma**	Normal<br>1+ Edema<br>2+ Edema<br>3+ Edema<br>4+ Edema<br>Scar<br>Other (specify)
**Endothelium**	Normal<br>1+ Folds<br>2+ Folds<br>3+ Folds<br>4+ Folds<br>Keratic<br>Guttata

	Other (specify)
**Iris- Rubeosis**	None<br>Mild<br>Moderate<br>Severe
**Lens**	Clear<br>Mild Opacity<br>Cataract<br>Intra ocular lens (IOL)
**Vitreous**	Normal<br>Degeneration<br>Posterior detachment<br>Hemorrhage<br>Opacity

Indirect ophthalmoscopy (Fundus Examination)

**Disc appearance**	Disc to cup ratio horizontal and vertical
**Disc appearance**	Healthy<br>Glaucomatous<br>Other (specify)
**Peripapillary Tissue**	Unremarkable<br>Abnormal (specify)
**Macula**	Unremarkable<br>Abnormal (specify)
**Vessels**	Unremarkable<br>Abnormal (specify)
**Periphery**	Unremarkable<br>Abnormal (specify)

Appendix 4 Trabeculectomy Standard-operating-procedure (SOP)

Pre-Operative Care

Patients will undergo a **washout-period according to their local hospital's regulations and standard medical care** prior to the trabeculectomy in order to discontinue treatment with any topical medication that is known or suspected to influence or induce inflammatory processes in the patient's eye.

In particular,

- topical **beta-blockers** and **prostaglandins** are to be discontinued (ideally **28 days** prior to the day of surgery).
- topical **alpha-2-agonists** and **carboanhydrase-inhibitors** are to be discontinued (ideally **7 days** prior to the day of surgery).
- other medication is to be discontinued according to its established pharmacological properties ideally with a sufficiently long wash-out period to ensure no effects on the trabeculectomy outcome.

For the duration of the wash-out phase, therapy can be substituted by topical, non-pressure-active **glucocorticoids** (e.g. fluorometholone, dexamethasone) and/or systemic **acetozolamide** (e.g. Diamox®), according to standard care exerted at the study center.

Trabeculectomy

The trabeculectomy procedure on study day 1 is to be done according to standard medical care established at the study center.

At the end of the surgical procedure 100 µl Mitomycin C (at a concentration of 200 µg/ml) are to be applied topically to the bleb.

Post-Operative Care

Hospitalization of the patient is required for two days after Day 1, but can be extended as medically indicated.

Post-operative care will follow established standard medical procedure at the study center.

Standard medication during the post-operative phase:

- Topical **antibiotics** for 5 days after trabeculectomy.
- Topical **glucocorticoids** (e.g. prednisolone acetate), starting at least 3 times daily, to be tapered over a four week period after trabeculectomy.
    - Use can be extended or intensified in case of corkscrew vessels of the bleb requiring further treatment.
- Topical **atropine or equivalent (e.g. cyclopentolate)**, starting 3 times daily, for 7 days after trabeculectomy.
    - Use can be extended or intensified in case of ocular hypertension requiring further treatment.

If seen as medically required by the Investigator, additional pharmacological treatment or other medical interventions can be used as necessary. In particular,

- **5-Fluorouracil (5FU)** depending on bleb status, by subconjunctival injection, at 5mg / 0.5ml dose, repeated as required.
- **Topical anti-glaucoma medication** (e.g. beta-blockers, alpha-2-agonists etc.) depending on IOP, if deemed necessary by the treating physician and if no reasonable alternative intervention is medically appropriate or has the patient's consent.

Non-pharmacological interventions (needling, suture lysis, re-operation) are to be done according to established medical standards as medically required.

Recording of Interventions
Any pharmacological treatment or **other medical intervention as listed above** during the trial, even if adherent to this SOP or other medical guidelines**, has to be reported in source data and the CRF**; pharmacological interventions can be assessed as "interventions post trabeculectomy" in the sense of the corresponding secondary endpoint. This assessment should be conducted according the following guidelines:

- Pharmacological interventions after trabeculectomy used **according to hospital standard,** independent from the case's clinical development, must not be documented as intervention post trabeculectomy but **only as concomitant medication (**e.g. glucocorticoids, antibiotics and mydriatics.);
  (important: no standard interventions according to this definition are use of 5-FU, needling with 5-FU or MMC and topical anti-glaucoma medication etc.);

- Pharmacological interventions after trabeculectomy **to improve the outcome and/or to treat or reduce complications,** in addition to the hospital standard, must be assessed **as intervention post trabeculectomy** (e.g. such as use of 5-FU, needling with 5-FU or MMC, topical anti-glaucoma medication);

- Pharmacological interventions **for treatment of other events/diseases** not directly related to trabeculectomy must not be documented as post trabeculectomy intervention but **only as concomitant medication**.

Appendix 5 Intravitreal Injection SOP

IMP dosing is done by intravitreal injection during the trabeculectomy procedure.

Intravitreal injections will be done by a qualified member of the study team as per local institutional practice and regulations. Study centers are advised to perform injections under observation of the guideline “Empfehlung der Deutschen Ophthalmologischen Gesellschaft, der Retinologischen Gesellschaft und des Berufsverbandes der Augenärzte Deutschlands für die Durchführung von intravitrealen Injektionen (IVI), Stand April 2007”.

Appendix 6 Intra ocular pressure measurement using the Goldmann tonometer SOP

Measurements of intraocular pressure are done using a slit lamp equipped with a Goldmann applanation tonometry extension. Setup of the equipment is to be done according to the manufacturer manual's specifications.

The right eye is always tested first. Two or three consecutive measurements are done to determine the intraocular pressure as detailed below. Each measurement is recorded in the CRF.

Measurement procedure:

Both of the patient's eyes will be anesthetized by applying e.g. 2-3 drops of an anesthetic within 30 seconds. Then a fluorescein paper strip is placed near the lateral canthus in the lower conjunctival sac. Once the lacrimal fluid is sufficiently colored, the paper strip is removed. Alternatively, pre-mixed fluorescein/anesthetic eye drops can be used. The same technique should be used each time.

The patient presses his head firmly against the chin and forehead rests. If necessary, a headband can be used for fixation of the position. The patient must look straight forward and will be reminded to keep his eyes open wide and to breathe normally during the examination. If it is necessary to hold the eyelids open by hand, care must be taken not to apply pressure to the globe.

Immediately before taking measurements, the patient should be instructed to blink briefly so that the cornea is sufficiently covered by fluorescein.

The prism is then gently brought into contact with the center of the cornea until the cornea's limbus takes on a bluish glow. Viewed through the slit lamp microscope, the semi-circular fluorescein bands need to be well-focused, centered horizontally and positioned vertically so their circumference is roughly equal above and below the horizontal dividing line. The width of the band should be about 1/10 of the applanation surface. If the bands are too narrow, more fluorescein needs to be instilled.

The pressure on the eye is increased by turning the tonometer measuring drum until the inner borders of both fluorescein bands just touch.

The tip is then removed from the cornea and the pressure reading on the device is recorded, rounded to the next highest integer.

The above procedure is then repeated for the same eye.

Determination of actual IOP measurement:

If the two measurements differ by 2 mmHg or less, the average of both measurements becomes the actual IOP value at this time point.

If the two measurements differ by 3 mmHg or more, then a third measurement is conducted, and the median of the measurements becomes the actual IOP value at this time point.

The IOP in the left eye is then measured using the same technique.

Appendix 7 Heidelberg Retina Tomograph II (HRTII) Ophthalmoscopy Interpretation (German and English)

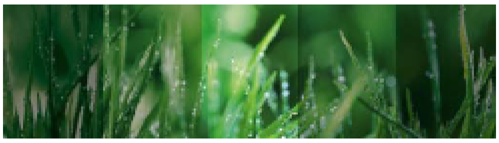

HEIDELBERG ENGINEERING

Interpreting the baseline examination in 60 seconds –
Intelligent combination of HRT information substantially enhances the sensitivity and specificity of the diagnostics

HRT

Reflectivity

A healthy nerve fibre layer demonstrates good reflectivity with striped radially emanating patterns.

If the picture quality and illumination are good, the damaged nerve fibre layer stands out due to its dull reflectivity.

Nerve fibre bundle defects present as sharply demarcated stripes compared to the healthy adjacent tissue with reduced reflectivity (dark) and usually emanate away from the temporal rim in a radial pattern.

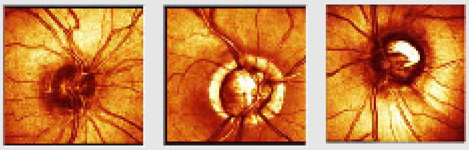

Good reflectivity | Dull reflectivity | Sharply demarcated nerve fibre bundle defect at 5:00 o'clock

Disc size

The disc is classified as small (less than approx. 2 mm²), normal or large (above approx. 3 mm²). Striking size differences between the right and the left eye are often interpreted as a risk factor (asymmetry).

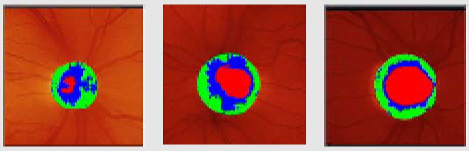

Small disc | Normal disc | Large disc

Cup shape

A vertically pronounced cup shape poses an additional risk factor.

Large discs with a horizontally pronounced cup tend to indicate a physiologically large optic nerve head and physiologic cupping.

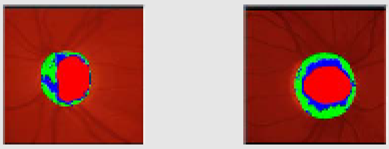

Vertically pronounced cup shape | Horizontally pronounced cup shape

Rim configuration

The ISNT rule for healthy optic nerve heads: The rim area of a healthy optic nerve head varies by sector, with the inferior being thickest, followed by the superior, nasal and temporal regions. Does the optic nerve head have a pathologically thin rim in the temporal sector?

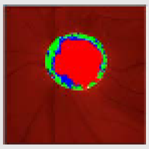

Optic nerve head with thin rim – ISNT rule not met

Assessing the RNFL height profile along the contour line

There should be a symmetrical double hump configuration of the height profile along the contour line at the disc margin. This is the height profile of the retinal nerve fibre layer. The height profile should intersect the mean height of the retina (0.00 line).

With large discs, the nerve fibres are distributed across a larger surface, and the height profile often does not reach the mean height of the retina.

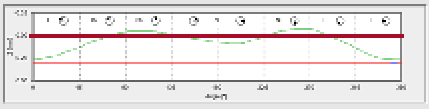

Symmetrical double hump configuration intersects mean height of retina (red)

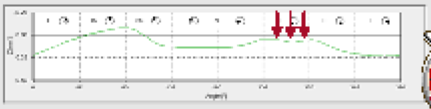

Double hump in inferior sector does not reach mean height of retina

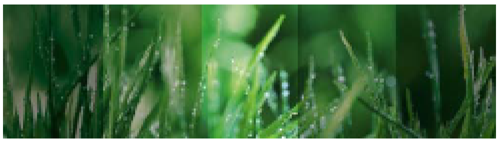

HEIDELBERG ENGINEERING

Rim volume

The rim volume should be at least 0.3 mm^3, regardless of disc size.

As with all stereometric parameters, in this case too, the p-value of the regression analysis is calculated and assessed using green, yellow and red symbols (Premium Edition only).

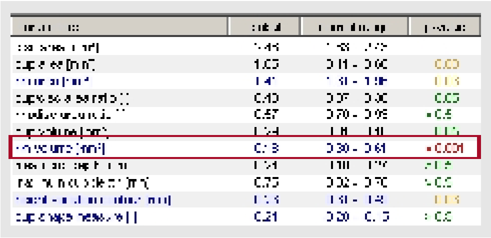

Cup shape measure (CSM)

The parameter for describing the cup shape (CSM) should be at least -0.2 for small and average discs or -0.1 (often larger or even slightly positive) for large discs. The more negative the value, the less suspicious the shape of the optic nerve head.

As with all stereometric parameters, in this case too, the p-value of the regression analysis is calculated and assessed using green, yellow and red symbols (Premium Edition only).

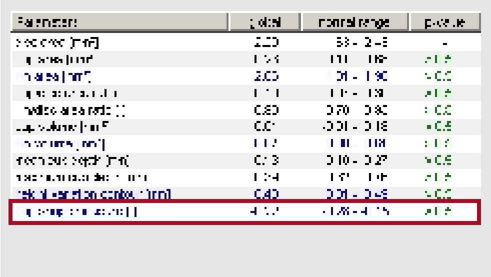

FSM and RB discriminant functions

The FSM (Frederik S. Mikelberg) and RB (Reinhard Burk) discriminant functions must be positive. In the case of large discs, if the FSM discriminant function is negative while the RB discriminant function remains positive, this frequently indicates a physiologically large optic nerve head and physiologic cupping.

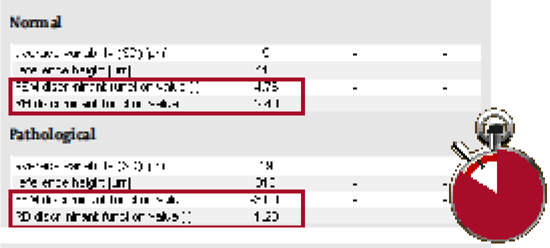

Moorfields Regression Analysis (MRA)

The MRA assesses the health of the rim relative to disc size. If the temporal, and specifically, the temporal inferior sector and the global MRA classification results are outside the normal limits (red x), this means that there is a substantially higher risk of glaucoma (OHTS Study). In the case of large or small discs, the correlation is less reliable.

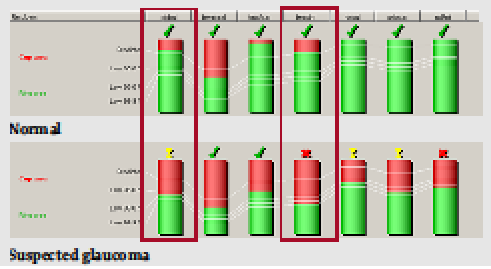

Glaucoma Probability Score (GPS)

(Premium Edition only)

The GPS automatically calculates the slope, size and depth of the optic nerve head and the curvature of the peripapillary retina. If the global result of the GPS analysis is outside the normal limits (red x), this means an elevated risk of glaucoma. In the case of large or small discs, the correlation is less reliable.

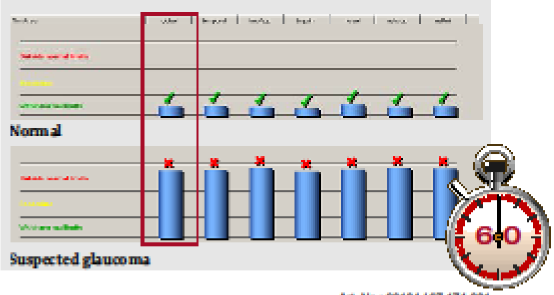

Art. No.: 99104 / 97 174-001

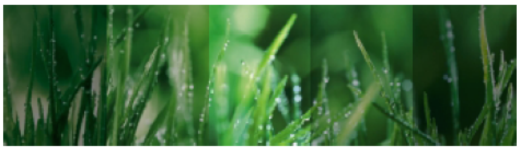

HEIDELBERG ENGINEERING

Interpretation der Erstuntersuchung in 60 Sekunden - Die sinnvolle Kombination der HRT-Informationen steigert Sensitivität und Spezifität der Diagnostik entscheidend

HRT

Reflektivität

Eine gesunde Nervenfaserschicht zeigt eine gute Reflektivität mit streifig radial laufenden Mustern.

Bei sonst guter Bildqualität und Ausleuchtung fällt die geschädigte Nervenfaserschicht durch ihre stumpfe Reflektivität auf.

Nervenfaserbündeldefekte erscheinen als gegenüber dem gesunden Nachbargewebe scharf abgegrenzte Streifen mit reduzierter Reflektivität (dunkler) und laufen radial meist vom temporalen Randsaum weg.

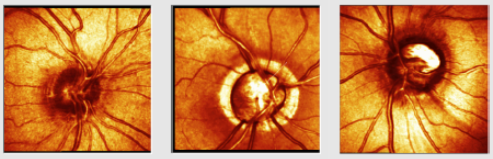

gute Reflektivität | stumpfe Reflektivität | scharf abgegrenzter Nervenfaserbündeldefekt bei 5 Uhr

Papillengröße

Einordnung der Papille als Mikro- (unter ca. 2 mm²), Makro- (ab ca. 3 mm²) oder Normalpapille. Auffällige Größenunterschiede zwischen rechtem und linkem Auge werden oft als Risikofaktor interpretiert.

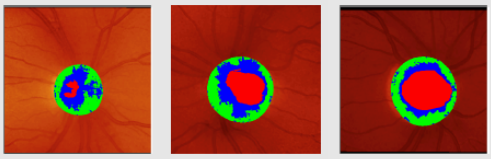

Mikropapille | Normalpapille | Makropapille

Exkavationsform

Eine vertikal betonte Exkavationsform ist ein zusätzlicher Risikofaktor.

Große Papillen mit einer horizontal betonten Exkavation weisen eher auf eine physiologische Makropapille hin.

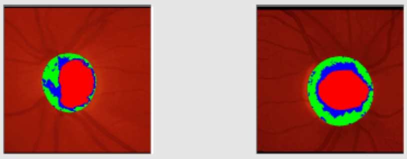

vertikale Betonung | horizontale Betonung

Randsaumkonfiguration

ISNT Regel für gesunde Papillen: Der inferiore Randsaum ist am breitesten, gefolgt vom superioren, nasalen und temporalen Randsaum. Ist die Papille temporal pathologisch randständig?

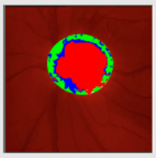

randständige Papille – ISNT Regel nicht erfüllt

Bewertung des Höhenprofils entlang der Konturlinie

Es sollte eine symmetrische Doppelhügelkonfiguration des Höhenprofils entlang des zuvor abgegrenzten Papillenrandes vorliegen. Das Höhenprofil sollte das mittlere Netzhautniveau (0.00 -Linie) schneiden.

Bei Makropapillen verteilen sich die Nervenfasern über eine größere Fläche, und das Höhenprofil erreicht das mittlere Netzhautniveau oft nicht.

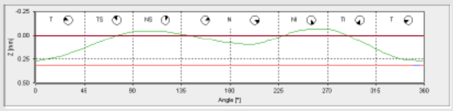

symmetrische Doppelhügelkonfiguration schneidet mittleres Netzhautniveau (rot)

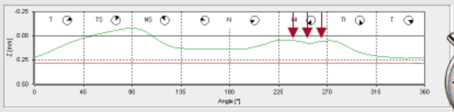

Doppelhügel erreicht inferior nicht das mittlere Netzhautniveau

Randsaumvolumen

Das Randsaumvolumen sollte unabhängig von der Papillengröße mindestens 0,3 mm³ betragen.

Wie für alle stereometrischen Parameter wird auch hier der p-Wert der Regressionsanalyse berechnet und mit Hilfe der Ampelfarben bewertet (nur Premium Edition).

Parameters	global	normal range	p-value
disc area [mm²]	2.46	1.63 - 2.43	-
cup area [mm²]	1.05	0.11 - 0.68	0.03
rim area [mm²]	1.41	1.31 - 1.96	0.03
cup/disc area ratio [ ]	0.43	0.07 - 0.30	0.05
rim/disc area ratio [ ]	0.57	0.70 - 0.93	> 0.5
cup volume [mm³]	0.29	-0.01 - 0.18	0.05
rim volume [mm³]	0.18	0.30 - 0.61	< 0.001
mean cup depth [mm]	0.24	0.10 - 0.27	> 0.5
maximum cup depth [mm]	0.75	0.32 - 0.76	> 0.5
height variation contour [mm]	0.23	0.31 - 0.49	0.03
cup shape measure [ ]	-0.24	-0.28 - -0.15	> 0.5

Cup Shape Measure (CSM)

Die einheitenlose Kennzahl für die Form des Sehnervenkopfes CSM sollte mindestens -0,2 für Mikro- und Normalpapillen bzw. -0,1 (nicht selten größer oder gar leicht positiv) für Makropapillen betragen. Je negativer der Wert, um so unverdächtiger ist die Form des Sehnervenkopfes.

Wie für alle stereometrischen Parameter wird auch hier der p-Wert der Regressionsanalyse berechnet und mit Hilfe der Ampelfarben bewertet (nur Premium Edition).

Parameters	global	normal range	p-value
disc area [mm²]	2.29	1.63 - 2.43	-
cup area [mm²]	0.23	0.11 - 0.68	> 0.5
rim area [mm²]	2.06	1.31 - 1.96	> 0.5
cup/disc area ratio [ ]	0.10	0.07 - 0.30	> 0.5
rim/disc area ratio [ ]	0.90	0.70 - 0.93	> 0.5
cup volume [mm³]	0.01	-0.01 - 0.18	> 0.5
rim volume [mm³]	0.67	0.30 - 0.61	> 0.5
mean cup depth [mm]	0.13	0.10 - 0.27	> 0.5
maximum cup depth [mm]	0.39	0.32 - 0.76	> 0.5
height variation contour [mm]	0.43	0.31 - 0.49	> 0.5
cup shape measure [ ]	-0.22	-0.28 - -0.15	> 0.5

Diskriminanzfunktionen FSM und RB

Die FSM (Frederik S. Mikelberg) und die RB (Reinhard Burk) Diskriminanzfunktionen müssen positiv sein. Wird bei großen Papillen die FSM-Diskriminanzfunktion negativ, während die RB-Diskriminanzfunktion positiv bleibt, ist dies häufig ein Hinweis auf eine physiologische Makropapille.

normal

average variability (SD) [µm]	9	-	-
reference height [µm]	418	-	-
FSM discriminant function value [ ]	4.76	-	-
RB discriminant function value [ ]	2.40	-	-

pathologisch

average variability (SD) [µm]	19	-	-
reference height [µm]	316	-	-
FSM discriminant function value [ ]	-3.09	-	-
RB discriminant function value [ ]	-1.28	-	-

Moorfields Regressionsanalyse (MRA)

Die MRA bewertet die Randsaumfläche im Verhältnis zur Papillengröße. Liegt das temporale, speziell das temporal inferiore, ferner das globale MRA-Klassifikationsergebnis außerhalb normaler Grenzen (rotes Kreuz), bedeutet dies ein stark erhöhtes Glaukomrisiko (OHTS Studie). Bei Makro- oder Mikropapillen ist die Aussagekraft eingeschränkt.

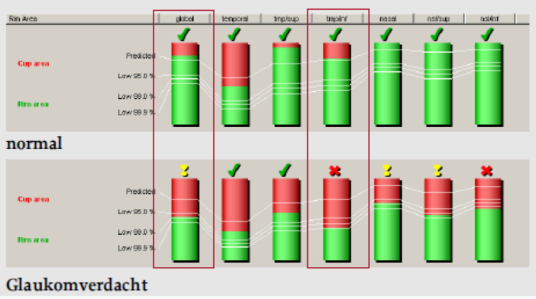

Glaucoma Probability Score (GPS)

(nur Premium Edition)

Der GPS bewertet vollautomatisch Steilheit, Größe und Tiefe des Sehnervenkopfes bzw. die Krümmung der peripapillären Retina. Liegt das globale Ergebnis der GPS-Analyse außerhalb normaler Grenzen (rotes Kreuz), bedeutet dies ein erhöhtes Glaukomrisiko. Bei Makro- oder Mikropapillen ist die Aussagekraft eingeschränkt.

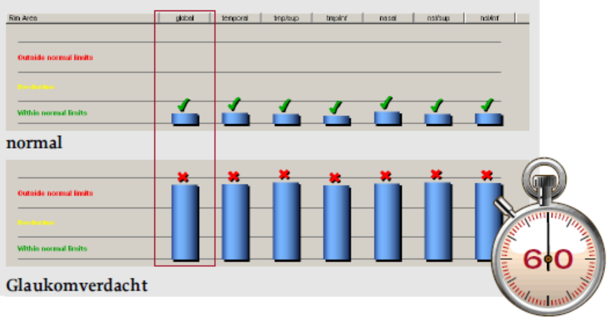

Appendix 8 NCI CTCAE classification

Ref: **CTCAE - EVS - National Institutes of Health**

The English version of the NCI CTCAE will be provided in booklet form to each study site.
