# Supplementary material for: First-in-human phase I study of ISTH0036, an antisense oligonucleotide selectively targeting transforming growth factor beta 2 (TGF-β2), in subjects with open-angle glaucoma undergoing glaucoma filtration surgery
Source: PLoS One. 2017 Nov 30;12(11):e0188899. doi: 10.1371/journal.pone.0188899 (PMC5708654; doi:10.1371/journal.pone.0188899)
Supplement: S3 File — (PDF) [file pone.0188899.s003.pdf]

Intraocular pressure prior to surgery and on Day 43 and Day 85 by patient

		IOP (mmHG)		
Dose Level (DL)	Patient	IOP (presurgical)	Day 43/ Week 6	Day 85/ Week 12
DL1	1	24	9.5	10.5
	2	58	11	12.5
	3	27	9	6
DL2	1	14	13	12
	2	23	17	21.5
	3	33	4	9
DL3	1	23.5	3.5	4
	2	15	4	6
	3	23	9	7.5
DL4	1	43.5	9.5	7.5
	2	26	5	8.5
	3	17	8	7.5
