# Supplementary figures and images for: First-in-human phase I study of ISTH0036, an antisense oligonucleotide selectively targeting transforming growth factor beta 2 (TGF-β2), in subjects with open-angle glaucoma undergoing glaucoma filtration surgery
Source: PLoS One. 2017 Nov 30;12(11):e0188899. doi: 10.1371/journal.pone.0188899 (PMC5708654; doi:10.1371/journal.pone.0188899)

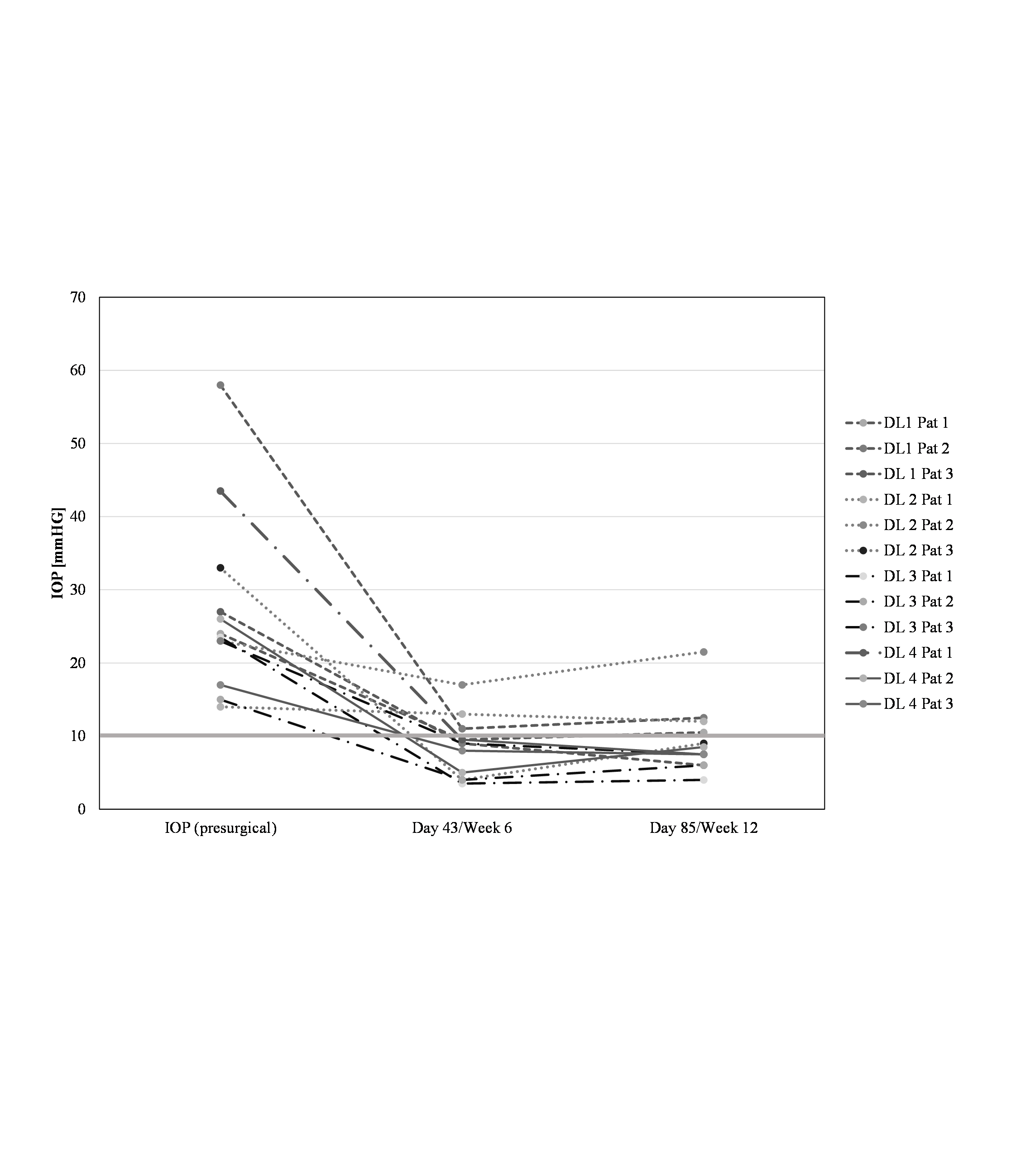

Supplement: S1 Fig — (TIF) [file pone.0188899.s004.tif]
